# Organization and evolution of *hsp70 *clusters strikingly differ in two species of Stratiomyidae (Diptera) inhabiting thermally contrasting environments

**DOI:** 10.1186/1471-2148-11-74

**Published:** 2011-03-22

**Authors:** David G Garbuz, Irina A Yushenova, Olga G Zatsepina, Andrey A Przhiboro, Brian R Bettencourt, Michael B Evgen'ev

**Affiliations:** 1 Engelhardt Institute of Molecular Biology, Russian Academy of Sciences, Moscow 119991, Russia; 2 Zoological Institute, Russian Academy of Sciences, St. Petersburg 199034, Russia; 3 Alnylam Pharmaceuticals, 300 Third St. Cambridge, MA 02142, USA; 4 Institute of Cell Biophysics, RAS, Pushchino, Moscow region,142290, Russia

**Keywords:** Diptera, *hsp70 *gene cluster, thermal adaptation, concerted evolution, Stratiomyidae

## Abstract

**Background:**

Previously, we described the heat shock response in dipteran species belonging to the family Stratiomyidae that develop in thermally and chemically contrasting habitats including highly aggressive ones. Although all species studied exhibit high constitutive levels of Hsp70 accompanied by exceptionally high thermotolerance, we also detected characteristic interspecies differences in heat shock protein (Hsp) expression and survival after severe heat shock. Here, we analyzed genomic libraries from two Stratiomyidae species from thermally and chemically contrasting habitats and determined the structure and organization of their *hsp70 *clusters.

**Results:**

Although the genomes of both species contain similar numbers of *hsp70 *genes, the spatial distribution of *hsp70 *copies differs characteristically. In a population of the eurytopic species *Stratiomys singularior*, which exists in thermally variable and chemically aggressive (hypersaline) conditions, the *hsp70 *copies form a tight cluster with approximately equal intergenic distances. In contrast, in a population of the stenotopic *Oxycera pardalina *that dwells in a stable cold spring, we did not find *hsp70 *copies in tandem orientation. In this species, the distance between individual *hsp70 *copies in the genome is very large, if they are linked at all. In *O. pardalina *we detected the *hsp68 *gene located next to a *hsp70 *copy in tandem orientation. Although the *hsp70 *coding sequences of *S. singularior *are highly homogenized via conversion, the structure and general arrangement of the *hsp70 *clusters are highly polymorphic, including gross aberrations, various deletions in intergenic regions, and insertion of incomplete *Mariner *transposons in close vicinity to the 3'-UTRs.

**Conclusions:**

The *hsp70 *gene families in *S. singularior *and *O. pardalina *evolved quite differently from one another. We demonstrated clear evidence of homogenizing gene conversion in the *S. singularior hsp70 *genes, which form tight clusters in this species. In the case of the other species, *O. pardalina*, we found no clear trace of concerted evolution for the dispersed *hsp70 *genes. Furthermore, in the latter species we detected *hsp70 *pseudogenes, representing a hallmark of the birth-and-death process.

## Background

The heat shock response is a universal phenomenon, and heat shock proteins (Hsps) form the most ancient and sophisticated cellular defense system present in all living organisms [1,2]. The Hsps are broadly classified on the basis of their molecular weights and functions into distinct families, with the Hsp70 family being the best studied and among the most important for cell protection [3,4]. Though the heat shock response is a universal phenomenon, various organisms even within the taxonomic groups of relatively low rank (*e.g.*, order, family) often evolve different strategies to adapt to aggressive environments [4-8].

The mechanisms providing whole body adaptation of animals to various adverse conditions, including extreme thermal environments, have long attracted the attention of ecologists. After the description of Hsps in *Drosophila *and other organisms [1,9], we undertook systematic studies to establish a possible correlation between the general pattern of Hsp synthesis and whole-body adaptation to extreme conditions [7,10,11]. It is noteworthy that the predominant fraction of investigations on the stress response have largely (but not entirely) been previously performed on a small number of model organisms in the laboratory and, therefore, have limited value in an ecological context. Keeping this in mind we usually included in our investigation related species or geographical strains from nature, in particular those inhabiting biotopes or regions with strikingly contrasting average temperatures [8,11-14]. Using this approach, we, as well as other research groups, have accumulated abundant data showing that animals have evolved a variety of strategies to adjust their heat shock systems to changing environmental conditions [4,7]. Long-term and large-scale studies of the *Hsp*s gene system in species and geographical strains that differ in the temperature of habitats, performed in various laboratories, have disclosed a few basic patterns in the functioning and evolution of this system in organisms living under adverse conditions [4,5,11,15-17].

In the course of these studies, it was repeatedly demonstrated that noticeable cellular levels of Hsps and/or corresponding RNA under normal physiological temperatures represent one of the most common adaptations to stressful conditions described in various organisms, including certain species of lizards, flies and ants [11,15,18].

In particular, in order to investigate how organisms have evolved to occupy contrasting habitats, we compared four species of Stratiomyidae (Diptera) whose larvae develop in naturally varying (semi)aquatic habitats, including highly aggressive conditions of hot volcanic springs [8]. Previously we have demonstrated that larvae of all these species, independently of their thermal history, show a high level of inducible Hsp70 under "normal" physiological conditions (constitutive or chronic expression), and that the level increases only 2 - 3 fold after a 30 minutes heat shock treatment and 24 hours of recovery. Larvae of these species are able to survive exceptionally heavy heat shock treatments (46 - 48°C), which are lethal to most of other dipteran species studied so far [19-21].

We also demonstrated that *Oxycera pardalina *Meigen, 1822, a Stratiomyidae species confined to cold and thermally constant environments, shows the highest relative heat-shock tolerance (*i.e. *can survive the largest shift in temperature as compared to that in its normal habitat). Whether and how the evolutionary alteration of Hsp70 expression and thermotolerance among the Stratiomyidae species involves changes to the *hsp70 *genes themselves remain unanswered questions.

Here we report the results of molecular analyses performed to compare the general arrangements and sequence of *hsp70*-containing loci in two Stratiomyidae species from contrasting thermal environments, *O. pardalina *and *Stratiomys singularior *Harris, 1776. For this purpose, we cloned and sequenced member genes of the *hsp70 *gene superfamily from the two species compared.

Our studies have demonstrated that the population of *S. singularior *that inhabits an area with very instable extreme larval habitats and strongly variable conditions, is characterized by a compact *hsp70 *cluster of 5 converting *hsp70 *genes. Contrastingly, the stable and constantly cold habitat-dwelling *O. pardalina *exhibits quite different distribution and spacing of *hsp70 *copies in the genome.

## Results

### General organization of hsp70 clusters in *S. singularior *and *O. pardalina*

**Figure 1:**
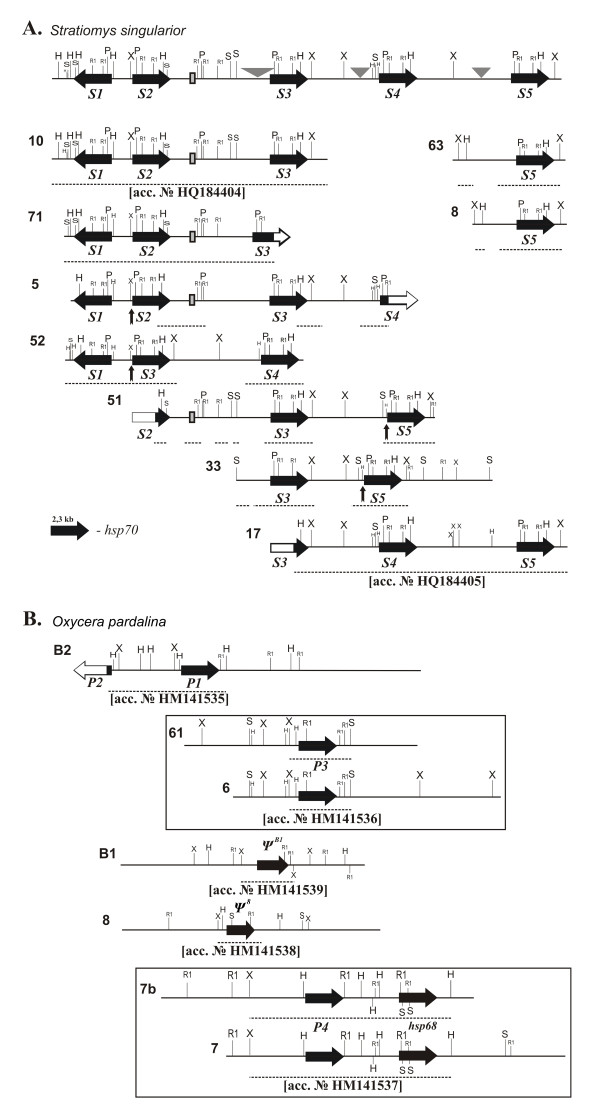
**Nomenclature and organization of *hsp70 *genes in *S. singularior *(A) and in *O. pardalina *(B) genomes**. Deduced arrangement of *hsp70 *cluster in *S. singularior *is given at the top. Names of the clones (phages) are given on the left. Individual *hsp70 *genes are represented by thick filled arrows with the names given underneath. The sequenced fragments are indicated by dotted lines underneath. Accession numbers for the deposited sequences are provided underneath the correspondent sequences. Vertical black arrows underneath phages 51, 52 and 33 indicate the sites of presumptive interlocus recombination. A typical copy of *Hsp70 *gene in a scale is given at the left side. Triangles indicate the sites where deletions have been detected in a few phages. Filled rectangle show the location of a fragment of Mariner transposable element. H - *HindIII*, P - *PstI*, R1 - *EcoRI*, S - *SalI*, X - *XbaI*.

The detailed organization of *hsp70 *genes in *S. singularior *and *O. pardalina *emerges from the subcloning and sequencing of overlapping lambda clones isolated from genomic libraries (Figures 1A and 1B). We have isolated twenty lambda clones from a *S. singularior *genomic library, and after preliminary restriction analysis have chosen nine clones which apparently contain one or several complete *hsp70 *genes (Figure 1A). From these nine phages we have isolated and described four *hsp70 *copies in tandem orientation (*hsp70S2-S5*) and a fifth copy in inverse orientation (*hsp70S1*). Genes *S2 *- *S5 *are not identically spaced, and intergenic regions vary: approximately 4.7 kb between *hsp70S3 *and *S4*, 5.3 kb between *hsp70S4 *and *S5*, 5.9 kb between *hsp70S2 *and *S3*, and 1.2 kb between *hsp70S1 and S2*. The whole *hsp70 *cluster of *S. singularior *was sequenced, and surprisingly we failed to detect any significant homology among the intergenic regions between individual *hsp70 *genes.

Characteristically, we detected various polymorphisms within the same intergenic regions isolated from individual clones. These polymorphisms include the insertion of a fragment (314 bp in length) of *Mariner *transposon in the *hsp70S2-hsp70S3 *intergenic region detected in all four phages containing these genes (phages 10, 71, 52 and 5). Furthermore, *hsp70 *copies in *S. singularior *cluster are subject to gross rearrangement, and although their order in most phages is *S2, S3 *and *S4*, in phages 33 and 51 the *S5 *gene is located after *S3*, while in phage 52 an inverted pair is formed by *S1 *and *S3 *genes (Figure 1A).

Interestingly, we detected deletions in almost all the *hsp70 *intergenic regions. In phage 8, there is a 1 kb deletion in the *S4 *- *S5 *region, phage 71 contains a 2 kb deletion in the *hsp70S2 - hsp70S3 *region, and phage 33 contains a 2kb deletion in the *S3 *- *S5 *intergenic region. In phage 71, a deletion in the 5'-flank of *hsp70S3 *includes the beginning of the transcribed region and essential heat shock regulatory elements; this deletion may make this *hsp70S3 *allele noninducible (Figure 1A).

The general organization of the *hsp70 *genes in *O. pardalina *characteristically differs from that of *S. singularior *(Figure 1). Preliminary Southern blot analysis of eleven lambda clones isolated from *O. pardalina *genomic DNA and carrying *hsp70 *sequences showed that most of the cloned fragments contained only a single *hsp70 *copy (data not shown). This implies that most *hsp70 *copies in this species, with two prominent exceptions described below, are unlinked or at least located at significantly larger distances from each other than the *hsp70 *genes in *S. singularior*. We performed a detailed restriction analysis of seven lambda clones isolated from the *O. pardalina *genomic library, which contained the complete sequences of one or two *hsp70 *family copies (Figure 1B); the other four phages were excluded from the detailed analysis, because they contained only fragments of *hsp70 *genes and did not carry overlapping sequences, judging by cross hybridization with their labeled terminal fragments (data not shown).

Phage B2 contains one complete copy of *hsp70 *(*P1*) and a 5'-fragment of another *hsp70 *copy (*P2*) in inverse orientation, apparently cut by *Sau3A *in the process of cloning (Figure 1B). Although we were able to detect two copies of *hsp70 *in inverse orientation in *O. pardalina *in phage B2, even in this case, the distance between these copies was 4.6 kb, *i.e. *four times as large as the correspondent intergenic region between the inverted *S1 *and *S2 *genes in *S. singularior *(Figure 1 A, B). The 5'-flanking regions of all *hsp70 *copies in *O. pardalina *exhibit extended homology upstream from transcription start (up to 650 bp including 5'-UTRs), while in *S. singularior*, 5'-flanking homology in the correspondent intergenic region is evident only within approximately 200 bp. This implies that in *O. pardalina*, the *hsp70 *genes undergo concerted evolution (*i.e. *interlocus recombination) in blocks containing more than 650 bp of 5'-flanking sequences, while in *S. singularior *concertedly evolving blocks include less than 200 bp of 5'-flanking sequences.

Figure 1B depicts four phages containing essentially the same *hsp70 *genes (*i.e. *clones 61 and 6 and 7b and 7). These phages are included into this figure to illustrate that in contrast to *S. singularior, hsp70 *genes in *O. pardalina *are not arranged in tight clusters, and indeed may be entirely unlinked. It is noteworthy that sequencing of *hsp70 *genes and intergenic regions from these phages did not reveal any differences. Hence, we conclude that phages 61 and 6 and 7b and 7 contain DNA from full sibs.

In *O. pardalina*, we observed two *hsp70 *pseudogenes (Figure 1B). The pseudogene located in phage B1 is 89% homologous to *hsp70P1 *and contains several short deletions resulting in ORF shifts. The other pseudogene, isolated from phage 8, is also homologous to *hsp70P1 *(86%) and represents a highly rearranged copy. It contains a long deletion (306 bp) and inversion (677 bp) in the coding region of the gene. This pseudogene does not contain the first ATG codon. Both pseudogenes lack HSE elements and fail to exhibit any homology with functional *hsp7*0 genes of *O. pardalina *in the 5'-flanking regions. The *O. pardalina hsp70 *pseudogenes likely arose by retroposition, as suggested for similar duplicated *hsp70 *genes and pseudogenes in *Drosophila *[22,23]. Clones 7 and 7b contain homologous stretches of DNA carrying two tandem copies of genes belonging to the *hsp70 *family, located within a 3.5 kb interval. Interestingly, one copy is the inducible *hsp70P4*, and the second copy is probably *hsp68 *(see below). This is the first case to our knowledge when *hsp70 *and *hsp68 *genes were found to be so closely linked. Therefore, the detailed analysis of genomic libraries enabled us to describe five potentially functional *hsp70 *genes in *S. singularior *and five genes belonging to the *hsp70 *family, including a presumptive *hsp68 *gene, in *O. pardalina*. In order to make sure that we cloned and sequenced all or at least most of the *hsp70 *genes from the species compared, we performed a conventional Southern blot analysis using genomic DNA isolated from both species.

### Southern analysis of *S. singularior *and *O. pardalina *genomic DNA

**Figure 2:**
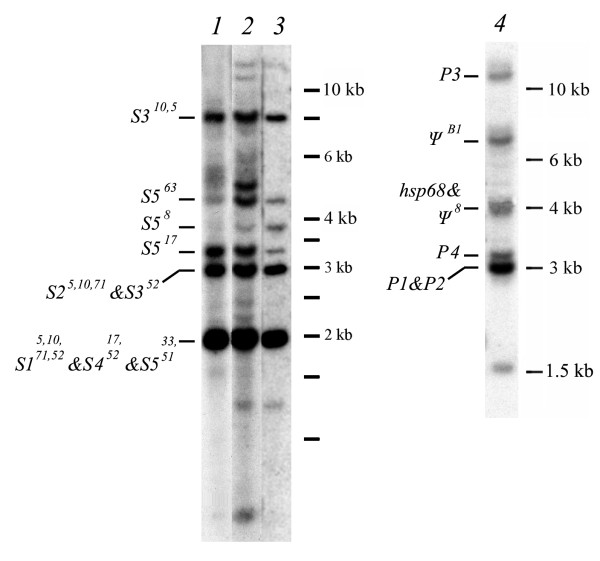
**Southern blot analysis of *HindIII *restricted genomic DNA isolated from *S. singularior *(lanes 1, 2, 3) and *O. pardalina *(lane 4)**. Numbers on the right indicate the size of the markers in kb. Lane 1 - *S. singularior *DNA isolated from twenty larvae hybridized with 5' PCR fragment of *S. singularior hsp70 *gene (1 - 538 bp PCR fragment of complete cDNA copy); 2 - *S. singularior*, DNA isolated from twenty larvae with 3'-PCR fragment of *S. singularior hsp70 *gene (1250 - 2340 bp PCR fragment of complete cDNA copy); 3 - *S. singularior *DNA isolated from five larvae with 3'-PCR fragment of *S. singularior hsp70 *gene (1250 - 2340 bp PCR fragment of complete cDNA copy); 4 - *O. pardalina*, DNA isolated from twenty larvae hybridized with PCR fragment included 298 - 1851 bp of complete *O. pardalina hsp70P1 *gene. Presumptive correspondence of individual *hsp70 *family genes (according to sequencing data) to the hybridizing bands is indicated on the left of the lanes.

Figure 2 depicts the results of Southern blot analysis, performed to obtain independent data on the number of *hsp70 *copies in the genomes of the two species studied. Restriction digests of both species' DNA were hybridized to a probe specific for the 5'- and 3'-regions of the *S. singularior hsp70 *coding sequence. The data depicted in Figure 2 confirm the results of the genomic libraries analysis described above, and show that the *S. singularior *genome apparently contains five copies of *hsp70*, while *O. pardalina *contains four *hsp70 *copies. Moreover, based on sequencing data we were able to establish the correspondence between individual *hsp70 *copies and hybridizing bands (see below and Figure 2). Thus, we have cloned and sequenced most if not all copies of *hsp70 *genes present in the genomes of both species. The additional minor bands seen in genomic DNA of *S. singularior *isolated from different numbers of larvae (Figure 2) may be explained by restriction site polymorphism or polymorphism in the structure of *hsp70 *genes *per se *and/or their flanking regions.

Preliminary 3'-RACE analysis of cDNA homologous to heat shock inducible RNA isolated from both species based on 3'-UTR heterogeneity in general has confirmed our conclusions about the *hsp70 *copy number estimated for both species, and indicates that all *hsp70 *copies isolated with the exception *O. pardalina *pseudogenes are apparently expressed, *i.e. *represented in the population of cDNA clones sequenced.

### Sequence analysis of hsp70 promoters and regulatory elements in *O. pardalina *and *S. singularior*

**Figure 3:**
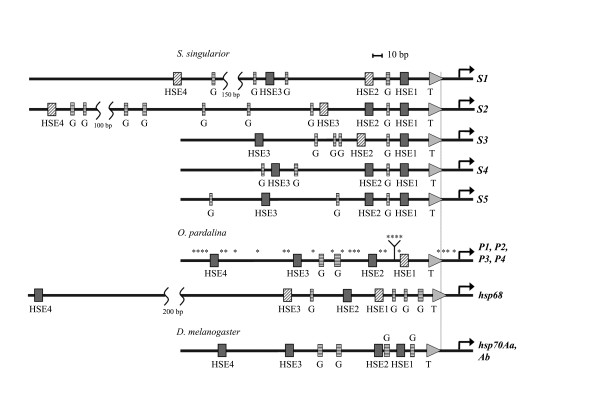
**General arrangement of promoter regions of *S. singularior *and *O. pardalina***. The locations of HSEs are indicated by filled horizontal rectangles. TATA-boxes are indicated by triangles (T). The shaded rectangles indicate the positions of non-canonical HSEs. Vertical rectangles indicate the positions of GAGA sites (G). The black bended arrows mark the transcription start and direction. D. melanogaster *hsp70 *promoter (from 70A region) is given at the bottom for comparison. Asterisks above the *O. pardalina *regulatory region mark single nucleotide polymorphisms.

Sequence analysis revealed all components of *hsp70 *regulatory machinery in the 5'-UTR regions of most *hsp70 *genes of both species compared. The results of comparing promoter regions in both species are depicted in Figure 3. The general structure of the *Drosophila melanogaster hsp70 *promoter is given for comparison.

We aligned the promoters of the *hsp70S *genes separately, since divergence among the genes becomes significant at approximately 40 bp upstream of the TATA signal (See Additional files 1, 2, 3, 4 and 5: Figures S1, S2, S3, S4 and S5 for individual alignments of the *S. singularior hsp70 *gene promoters). In *S. singularior*, promoters of individual *hsp70 *genes vary (Figure 3). Four candidate heat shock elements ("HSEs") are evident in the promoters of *hsp70S1 *and *hsp70S2*. In *hsp70S3, hsp70S4*, and *hsp70S5*, only three candidate HSEs are observed. Additionally, the promoters of *hsp70S3, S4*, and *S5 *contain indels and rearrangements. *hsp70S3^52 ^*contains a repetitive 39 bp insertion upstream of the TATA signal that ablates all but the most proximal of the HSEs (See Additional file 3: Figure S3). *hsp70S5^33 ^*has a similar type of 60 bp insertion upstream of the TATA signal, flanked by an *hsp70S4*-derived conversion tract (See Additional file 5: Figure S5). Whether these indels and rearrangements modulate *hsp70 *expression, as has been observed for *Drosophila hsp70 *genes [24,25], remains to be tested. Interestingly, in the same phages (52, 51 and 33) we observed gross rearrangements of the whole cluster (*i.e. hsp70S3 *in the place of *hsp70S2 *in phage 52, and *hsp70S5 *in the place of *hsp70S4 *in phages 51 and 33). The insertions of repetitive DNA thus likely represent the footprints of transposable elements responsible for the reshuffling of the *hsp70 *cluster observed in these particular phages. Intralocus unequal recombination likely occurred, because the promoter region of gene *S3 *in phage 52 is highly homologous to gene *S2*, including conversion tracts. Similarly, the promoter region of gene *S5 *from phages 33 and 51 is practically identical to that of *S4*. The sites of presumptive recombination breaks are shown by vertical arrows in Figure 1A.

The transcription start point was found to be identical in both species studied (see Figure 3). We identified the *hsp70 *transcription start by 5'-RACE using RNA isolated after HS from the larvae of both species, as described in Methods.

In all *S. singularior hsp70 *genes, the distance between TATA and transcription start point (AGT) is 24 bp, and the start codon is located 230 bp further downstream. In this species, all *hsp70 *TATA-boxes are represented by 5'-TATATATA-3' nucleotides, and GAG or GAGA corresponds to the position of the presumptive GAGA-factor binding site.

We aligned approximately 660 bp of promoter sequence from the four *Oxycera hsp70 *genes. These promoters are 93% identical to one another, and each contains four candidate HSEs in homologous positions ranging from 27 - 28 to 222 - 224 bp upstream of the TATA signal, which is represented by sequence TATAAATA (Figure 3).

The promoter of presumptive *hsp68*, cloned from *O. pardalina*, also contains a canonical TATA-box (TATAAATG), several GAG sequences, and four HSEs (Figure 3).

It is evident that essential regulatory elements located in the promoter regions of the two species *hsp70 *genes (*i.e. *HSEs and GAG or GAGA) vary in number and spacing when comparing between genes and species. In general, the spacing of major regulatory elements (HSEs and GAGA) in *O. pardalina *is more similar to that of *D. melanogaster *(Figure 3). All the above supports that promoters of both Stratiomyidae species studied are functional heat-inducible promoters.

### Sequence analysis and evolutionary relationships of hsp70 transcriptional units in *O. pardalina *and *S. singularior*

*Oxycera pardalina*: We recovered the full sequence of one allele for each of the *hsp70P1, hsp70P2*, and *hsp70P4 *genes. These genes are intronless and contain a 1920 bp CDS.

The three whole CDSs are on average 98.4% identical, diverging by 20 - 27 silent and 5 - 10 replacement substitutions (See Additional file 6: Table S1 for numbers of silent and replacement fixed differences). The 5'-UTRs of the three intact *hsp70 *genes are 252 - 260 bp long and 91% identical (data not shown).

We recovered 325 - 340 bp of alignable sequence downstream of the three cloned intact *hsp70 *CDSs; sequence identity in this region is 77%. In contrast to the near identity of *hsp70P1, P3*, and *P4*, two *hsp70 *pseudogenes, denoted *ψhsp70^B1 ^*and *ψhsp70^8^*, bear numerous indels and substitutions and do not encode full-length proteins. The UTRs and promoter regions of both pseudogenes were fully sequenced and aligned. However, we found neither potential HSE and TATA boxes nor any significant homology between these regions (data not shown).

*Stratiomys singularior*: We sequenced multiple alleles of all five *hsp70 *genes: *hsp70S1*, 3 alleles; *hsp70S2*, 2; *hsp70S3*, 4; *hsp70S4*, 2; *hsp70S5*, 5. Alleles are named according to the clones from which they were obtained (Figure 1A). All alleles contain an intronless, 1917 bp CDS; no pseudogenes were recovered. The CDSs are more similar to one another than was seen in *O. pardalina*; an average of seven fixed differences distinguish any pair of *S. singularior hsp70 *CDSs (See Additional file 7: Table S2 for MK tests results), for an average pairwise identity of 99.6%. Comparing the *hsp70S *genes to one another via MacDonald-Kreitman style tests reveals that in all cases, the number of polymorphisms is much greater than the number of fixed differences, and that both polymorphism and divergence are similarly skewed towards silent substitutions (Additional file 7: Table S2). These results are consistent with concerted evolution and purifying selection.

The mechanism of concerted evolution among the *hsp70S *CDSs is likely gene conversion, observed for other insect *hsp70 *genes [22,23]. There are 50 single-nucleotide polymorphisms ("SNPs") shared among the *hsp70S *genes (black bars in Additional file 8, Figure S6). Considering only the silent polymorphisms, to avoid confounding any effects of positive or balancing selection, we examined the probabilities of shared SNPs arising among the *hsp70S *genes via independent mutation or gene conversion using the method of Rozas and Aguade [26]. Table 1 indicates that in all pairwise comparisons of *hsp70S *genes, we can reject the null hypothesis of independent mutation at *P *< 10^-5^.

**Table 1:** Conversion-mediated shared polymorphism at the *Stratiomys hsp70 *genes

Gene	S2				S3				S4				S5			
	*N*	***η***_**σ**_	ssSNPs	*p*	*N*	***η***_**σ**_	ssSNPs	*p*	*N*	***η***_**σ**_	ssSNPs	*p*	*N*	*η*	ssSNPs	*p*
S1	889	37	6 (+1)	**<10^-6^**	889	35	13 (+5)	**<10^-6^**	890	49	11 (+3)	**<10^-6^**	890	68	13 (+1)	**<10^-6^**
S2					888	30	6 (+2)	**<10^-6^**	889	45	5 (+0)	<10-5	890	70	8 (+0)	**<10^-6^**
S3									889	46	7 (+2)	**<10^-6^**	890	68	10 (+3)	**<10^-6^**
S4													891	74	17 (+0)	**<10^-6^**

*N*, total number of silent sites. η_s_, total number of silent mutations. ssSNPs, number of shared silent single-nucleotide polymorphisms (numbers in parentheses indicate additional number of shared replacement single-nucleotide polymorphisms; not considered in probability estimation). *p*, probability of ssSNPs arising via parallel mutation, estimated from hypergeometric distribution.

We recovered and aligned 262 bp of 5'-UTR sequence from all the *hsp70S *genes/alleles whose CDSs were completely sequenced, plus one additional allele for each of *hsp70S3, hsp70S4*, and *hsp70S5 *(Additional file 9: Figure S7). Only the first 15 bp of the *hsp70S3^71 ^*5'-UTR could be aligned; the UTR is disrupted by large blocks of repetitive sequences upstream of this position, consistent with insertion and/or rearrangement via transposition. Whether this rearrangement alters the expression of *hsp70S3^71 ^*is unknown. The homogeneity observed in the *hsp70S *CDSs diminishes in the 5'-UTRs. The 5'-UTRs are 82% identical overall; linkage disequilibrium is higher than in the CDSs, and four blocks of linked, polymorphic sites that are shared among genes are strongly suggestive of conversion tracts (See grey bars in Additional file 9: Figure S7). Seventeen individual SNPs are shared among multiple genes, again consistent with gene conversion (black bars in Additional file 9: Figure S7).

The 3'-UTRs of the *S. singularior hsp70 *genes are more divergent than those of *O. pardalina*. In *S. singularior *the distance of a typical PolyA signal (AATAAA) from the stop codon, determined by 3'-RACE, varies from 193 to 247 bp in individual *hsp70 *copies.

In *O. pardalina *the position of a candidate polyadenylation signal slightly varies in individual copies of *hsp70 *genes and falls 183 - 186 bp downstream of the stop codon.

**Figure 4:**
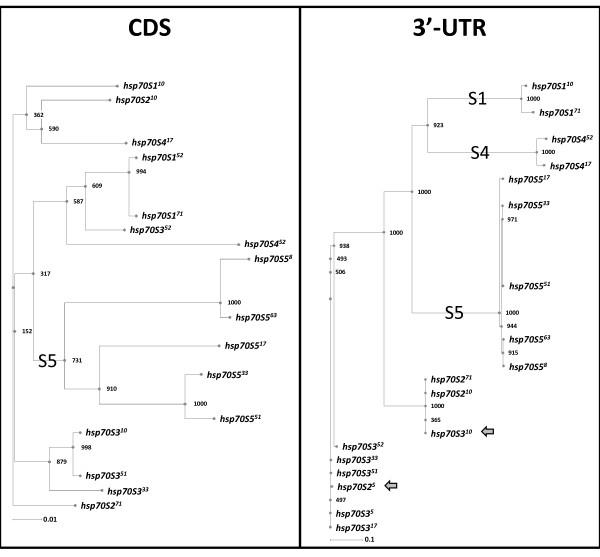
**Neighbor-joining phylograms of *Stratiomys hsp70 *coding sequences (left) and 3'-UTRs (right). Alleles named by phage number (superscript)**. Node labels indicate supportive number of 1000 bootstrap trials. Gene-specific clades labeled by gene abbreviation (*e.g.*, "S1" = *hsp70S1*). Rulers indicate genetic distance.

As for the 5'-UTRs, we sequenced 3'-UTRs of more alleles than just those for which we obtained full-length CDSs; more than 2 alleles of each *hsp70S *gene 3'-UTR were completely sequenced (Figure 1A). In contrast to the 5'-UTRs, which could all be aligned as one group, we aligned the 3'-UTRs of *hsp70S1, hsp70S4*, and *hsp70S5 *separately for visualization since their shared sequence is less than 100 bp long. Multiple candidate polyadenylation signals are found in all the 3'-UTRs. Only *hsp70S2 *and *hsp70S3 *share significant sequence identity. In general, the 3'-UTRs vary to a greater extent than both the CDSs and 5'-UTRs, with sequence identity <80% (See Additional files 10, 11, 12 and 13: Figures S8, S9, S10 and S11 for alignments of the 3'-UTRs). *Hsp70SS3^52 ^*has a deletion eliminating most of the 3'-UTR, approximately 47 bp downstream of the stop codon; the effects of this deletion were not tested but are consistent with rearrangements outside the *hsp70 *CDSs observed in the 5'-UTR and promoter (see above, below). Only *hsp70S2 *and *hsp70S3 *show evidence of gene conversion in the 3'-UTR: in clone 5, *hsp70S2*'s 3'-UTR has been converted over at least 300 bp to exactly resemble the 3'-UTR of *hsp70S3 *(see grey bars in Additional file 11: Figure S9). Similarly, in clone 10, *hsp70S3*'s 3'-UTR has been converted with *hsp70S2*'s. These less frequent, larger, and unbroken conversion tracts, along with the overall higher degrees of divergence among and polymorphism within 3'-UTRs, indicate less homogenization via gene conversion. This apparent reduction in conversion is more pronounced than is observed in the 5'-UTRs, which show both large tracts and the multiple shared SNPs indicative of homogenizing gene conversion. The net effect of conversion among CDSs with divergence among 3'-UTRs is evident in comparative phylogenetic trees (Figure 4). In the CDS tree, only *hsp70S5 *resolves as a clade and is supported by a majority of bootstrap trials. In contrast, the 3'-UTR tree contains *hsp70S1, hsp70S4*, and *hsp70S5 *clades, all with strong bootstrap support, and only the wholly-converted *hsp70S2/hsp70S3 *alleles from clones 5 and 10 prevent the assemblage of *hsp70S2 *and *hsp70S3 *clades. Thus, the *hsp70S *CDSs evolve in concert; the 3'-UTRs evolve largely independently.

The overall pattern of conversion-mediated homogeneity among *hsp70 *that decays with distance from CDS, and polymorphic flanks that contain large indels, is consistent with duplicated *hsp70s *in *Drosophila*.

### Inducible proteins encoded by hsp70 family genes in *S. singularior *and *O. pardalina*

Most of the varying sites in *hsp70 *genes of both species are silent and, hence, the deduced Hsp70s at amino acid level are highly homologous. Thus *S. singularior *individual Hsp70s exhibit almost 97% homology, while their homology with *O. pardalina *and *D. melanogaster *Hsp70s reaches 93% and 82%, respectively. Furthermore, the homology between deduced individual Hsp70s of *O. pardalina *reaches 99%. Characteristically, most of the varying sites in both species are localized in the C-domain of Hsp70, which is expected based on the above-presented data and the results of other authors showing a higher level of divergence at the 3'-ends of *hsp70 *genes in *Drosophila *[27,23]. The molecular weights of individual *S. singularior *Hsp70s (deduced by Vector NTI) are 69.89 - 70.18 kD and pI 5.48 - 5.75.

In order to further characterize Hsp70 family members in the species compared, we have performed two-dimensional electrophoresis of total proteins from *O. pardalina *and *S. singularior *with subsequent immunoblotting with the 7FB antibody, which in *D. melanogaster *reacts only with inducible Hsp70s, and with the 7.10.3 antibody, which reacts with all members of the Hsp70 family in most Diptera species examined including Hsp68 [8].

**Figure 5:**
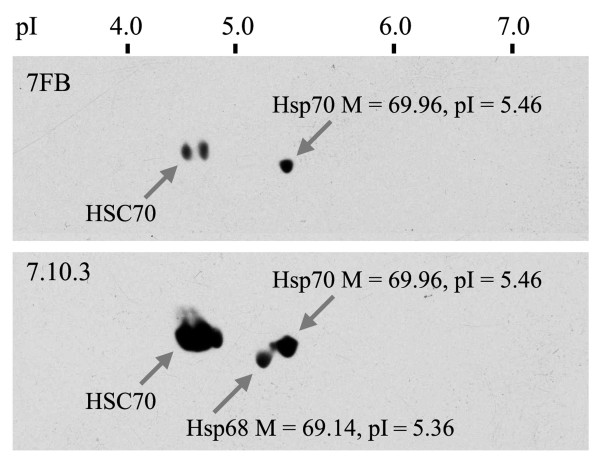
**The pattern of *O. pardalina *proteins as revealed by two-dimensional electrophoresis**. Western blotting of sections of 2D blots containing Hsp70 family members using 7FB (A) and 7.10.3 (B) antibodies. Constitutive (HSC70), inducible (Hsp70), and Hsp68 proteins are indicated by arrows.

The molecular weights of individual *O. pardalina *Hsp70 members deduced by Vector NTI (69.85 - 69.96 kD) and pI (5.45 - 5.46) vary and practically coincide with the results of 2D electrophoresis of *O. pardalina *proteins (Figure 5).

The 7.10.3, but not 7FB, antibody revealed one lower molecular weight protein, which characteristically has a slightly more acidic isoelectric point than the presumptive Hsp70 (Figure 5). We hypothesized that this particular protein corresponded to *D. melanogaster *Hsp68. Importantly, the molecular weight of this protein and pI revealed by 2D (69.1 and 5.4 correspondently) practically coincide with those of Hsp68 deduced basing on the presumptive *hsp68 *gene sequence located in phage 7 and 7b of *O. pardalina *(69.14 kD and 5.36).

Using Coomassie stained gels we have isolated spots of expected pI for the Hsp70 family members and performed fingerprinting and microsequencing analysis as described previously [8]. In both species, inducible proteins of interest are represented by a few isoforms, which are well separated on the gel and easy to isolate (Figure 5). After in-gel trypsin digestion and fingerprinting analysis using MALDI-ToF, the National Center for Biotechnology Information database was searched with the Profound search engine. This search has established that as expected the excised 70 kDa spots in both species correspond to members of the Hsp70 family previously described in various Diptera species (Additional file 1, Figure S12).

The predicted Hsp70 polypeptide of *S. singularior *and *O. pardalina *consists of 638 a.a. and 639 a.a., respectively, and presents all classic Hsp70 family signatures, including the presence of the serine residue in the ATPase domain (Additional file 14: Figure S12 for comparison with paralogs from several organisms) that characterizes the inducible HSP70 proteins of invertebrates [28]. We also detected a few amino acids residues specific for the both Stratiomyidae species and other highly thermotolerant organisms *e.g. Locusta migratoria *(Additional file 15: Figure S12). Conceptual translation showed that Stratiomyidae Hsp70 sequences had 72% identity with human Hsp70 and 84 - 85% identity with *D. melanogaster *Hsp70.

Our microsequencing data together with phylogenetic analysis and description of typical *hsps *promoters enable to conclude that all *hsp70 *genes isolated, with the prominent exception of the two *O. pardalina *pseudogenes, definitely represent inducible members of *hsp70 *family.

**Figure 6:**
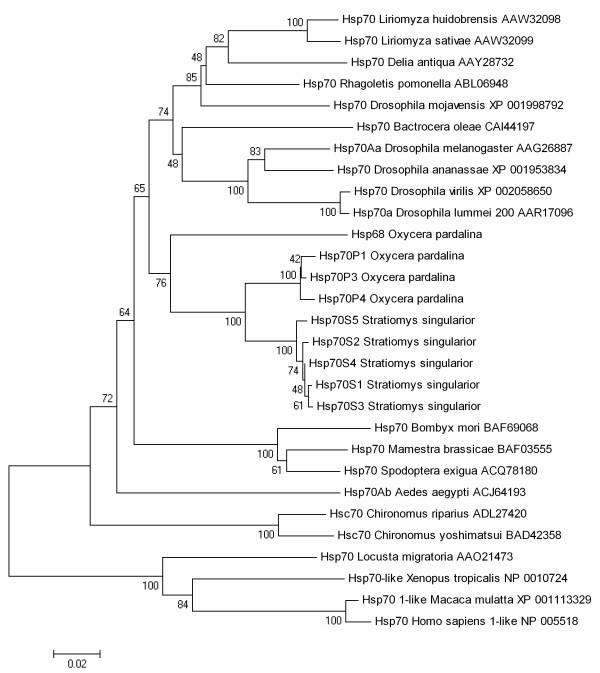
**Phylogenetic relationships of the Hsp70 proteins from *S. singularior, O. pardalina *and several other species**. Accession numbers are given after species name. The evolutionary history was inferred using the neighbor-joining method. The percentage of replicate trees, in which the associated taxa clustered together in the bootstrap test (500 replicates), are shown next to the branches. The tree is drawn to scale, with branch lengths in the same units as those of the evolutionary distances used to infer the phylogenetic tree. The evolutionary distances were computed using the Poisson correction method and are in the units of the number of amino acid substitutions per site. All positions containing gaps and missing data were eliminated from the dataset.

Previously we have shown that all four Stratiomyidae species exhibit high thermotolerance, correlated with high constitutive cellular levels of Hsp70. Surprisingly, Northern hybridization experiments failed to demonstrate *hsp70 *expression under normal physiological conditions, and only using a highly sensitive RT-PCR technique we were able to detect a very weak leakage of *hsp70 *genes in the species studied [8]. Surprisingly, the accumulation of Hsps in the cells of these species after heat shock challenge continues for many hours after HS treatment and, therefore, inducible Hsp70s in these species are probably more stable in comparison with *Drosophila *and many other Diptera species [29]. Based on these data, it was of significant interest to compare the amino acid sequence of Stratiomyidae Hsp70s with the sequence of Hsp70s isolated from other animals. The results of this analysis are shown in Figure 6.

Roughly, the dendrogram reflects the accepted phylogenetic relationships between the taxa included [30], with some deviations. In Figure 6, species of Stratiomyidae (family belonging to the suborder Orthorrhapha of the order Diptera) form a separate cluster, as well as all species of another suborder, Cyclorrhapha (the families Drosophilidae, Agromyzidae, Tephritidae), with one exception, *Bactrocera oleae *(Tephritidae). These two branches were clustered together. Contrastingly, members of the "more primitive" suborder Nematocera (Chironomidae, Culicidae) have the much lower similarity to other Diptera; formally, they were grouped with the cluster (Lepidoptera + all other Diptera) and did not form a separate cluster (Figure 6).

Interestingly, heat shock proteins (Hsp70) from both Stratiomyidae species grouped with paralogous sequences from several insect species including the ones known to be highly thermotolerant such as *Bombyx mori *[10], *Locusta migratoria *[31] and *Drosophila mojavensis *[29]. However, although even a few mammalian Hsp70s were included into the tree, it is premature to speculate on any functional relatedness of these proteins because such speculation will require the inclusion and analysis of many more sequences.

## Discussion

In this paper we investigated the structure and general arrangement of *hsp70 *genes from two Stratiomyidae species from strikingly contrasting habitats.

According to field observations and measurements, *S. singularior *has highly variable and instable larval habitats in the arid steppe zone of the Crimea. Strong changes in the shore larval habitats of *S. singularior *were observed since 2005 to 2009, both within the season and between the years. These changes were related mostly to hydrological regime, *i.e. *varying precipitation and the water level of the lakes. Generally, in the periods of the higher water table, many larval habitats were flooded or strongly influenced by hypersaline lake water (mineralization reached 150 - 280‰ and higher). The decreasing water level of the lake coincided with a decrease of mineralization (up to 80 - 35‰ in some larval habitats); in these periods, the respective habitats were moistened mostly with groundwater of lower salinity. It should be also mentioned that the abundance of *S. singularior *larvae varied greatly along the shoreline and between the (micro)habitats. In favorable places, *e.g. *in some groundwater-fed pools above the lake water margin, the larvae reached high abundance (hundreds and thousands individuals per m^2^), but typically, they were completely absent or found only as single individuals along the most part of the shore.

It seems that in the study area, *S. singularior *is represented by a number of micropopulations mostly confined to some shore localities, which may be at a distance of several km from each other. Many of these favorable breeding places are temporary and completely dry out in drier and warmer years. However, in this case adult flies are able to cover the distance of several kilometers easily in search of new places for oviposition.

Therefore, our preliminary observations on the Crimean larval habitats of *S. singularior *testify to a variability in the conditions between the habitats, strong variation of conditions within the habitats occurring with time, together with the temporary nature of many habitats and their possible isolation from each other. All these features may result in a high polymorphism of *hsp70 *demonstrated in this particular species.

We demonstrated a high level of polymorphism by Southern blot hybridization with genomic DNA digests, depending on the number of the larvae taken for analysis (Figure 2). Furthermore, sequencing of several phages containing the same *hsp70 *genes revealed polymorphism in the intergenic regions including long deletions. In a few cases we detected deletions of entire individual *hsp70 *genes (in phages 52, 33 and 51), likely a result of unequal recombination.

These deletions may result from an evolutionary trend to make the *hsp70 *cluster in this species more compact. Previously we have described more compact organization of the *hsp70 *genes cluster in a more thermotolerant low-latitude species *Drosophila virilis *in comparison with the closely related higher-latitude *D. lummei *from the temperate climatic zone [13] and, therefore, the observed deletions may represent certain pattern. Alternatively, the deletions described in *S. singularior *may be due to a high overall rate of DNA loss in this species, a phenomenon previously demonstrated for two *Drosophila *species [32].

In contrast to the *S. singularior *biotope, the studied larval habitat of *O. pardalina *- a cold spring near St. Petersburg observed from 2004 to 2010 - was very stable in mineralization, temperature, water regime, and in other major conditions both within season and interannually. This spring and headwater stream seems to be an island habitat for the *O. pardalina *population. Another similar habitat is situated at about 3 km, and it is unlikely that *O. pardalina *adults migrate regularly between these localities. The abundance of larvae is not high, not exceeding 10 - 50 individuals per m^2 ^of semiaquatic shore substrate (moss etc.). Thus, habitats of the two species differ dramatically from each other not only in the average parameters such as temperature and mineralization, but also in the spatial organization and in the degree of variability in time.

Therefore, in the case of the latter species we have a comparatively small and probably highly homozygous population. Southern blot hybridization analysis (Figure 2) did not reveal any polymorphisms in *O. pardalina hsp70 *genes and, hence, confirms this conclusion. Furthermore, individual phages containing the same fragments of DNA did not exhibit any polymorphisms either in coding or in intergenic regions of the same *hsp70 *genes. Since these phages were isolated from two independently obtained genomic libraries they cannot contain the same DNA but rather likely represent highly homologous sibs.

Previously, when we investigated inbred laboratory strains of *D. virilis *and *D. lummei*, we also demonstrated apparent identity of the overlapping *hsp70*-containing clones, which is typical for highly homozygous strains [13].

In spite of strikingly different habitats, the larvae of all Stratiomyidae species studied have very high tolerance to elevated temperature in comparison with most of other Diptera species investigated [8]. Furthermore, although tolerances were in general correlated with the typical habitat temperatures of the species, even *O. pardalina *larvae inhabiting cold springs with constant temperature regime (5 - 10°C), significantly exceeded all *Drosophila *species in tolerance and exhibited a huge gap between the natural temperatures (5 - 10°C) and the critical temperature (43°C).

Importantly, larvae of all four Stratiomyidae species investigated have exhibited high constitutive levels of Hsp70 proteins which are induced only 2 - 3-fold after temperature elevation [8]. In this respect, Stratiomyidae species resemble *Locusta migratoria*, an insect species adapted to arid tropical and subtropical climate, where temperature frequently exceeded 40°C [31].

Additionally, in contrast to *Drosophila*, the accumulation of Hsp70 continues in the cells of the treated larvae of all Stratiomyidae species investigated for many hours after heat shock and plateaus only approximately 24 - 36 h after the treatment [8].

The results obtained may be explained by constitutive weak transcription (leakage) of *hsp70 *genes under normal physiological conditions, and possibly by comparatively higher stability of Hsps in Stratiomyidae species in comparison with other insect species studied.

In order to further characterize the molecular basis underlying this pattern of *hsp70 *expression and differences in thermotolerance in the Stratiomyidae and other Diptera, as well as between the Stratiomyidae species, we compared the general arrangement of *hsp70 *genes clusters of the two Stratiomyidae species from thermally different habitats. Surprisingly, the analysis revealed quite different organization of *hsp70 *genes in the two species compared.

Although the *hsp70 *copy number does not differ significantly between less tolerant *O. pardalina *and more thermotolerant *S. singularior*, the general organization of *hsp70 *genes is divergent in these two species. While in *S. singularior *all *hsp70 *genes are confined to one genomic section of about 29 kb in length, in *O. pardalina *most of *hsp70 *genes are scattered in the genome and apparently located rather far from each other if linked at all. However, in both species we have found two *hsp70 *copies in an inverted orientation (a fundamental and probably ancient *hsp70 *gene unit) [22]. Furthermore, in *O. pardalina *we cloned a fragment carrying one copy of inducible *hsp70 *and one copy of presumptive *hsp68 *gene in tandem orientation separated by only 3.5 kb.

Previously we described two *hsp68 *copies in inverted orientation in species of the *virilis *group of *Drosophila *[13]. We speculated that *hsp68 *in these species arose by duplication of a basic unit of *hsp70 *genes typically arranged in Diptera species in inverted orientation. However, in the *virilis *group species *hsp70 *and *hsp68 *are located in the same chromosome but are separated by a large distance [13]. Presumptive *hsp68 *gene of *O. pardalina *is closely linked to *hsp70 *gene (Figure 1B) and exhibits higher homology with *hsp70 *genes of this species than with *hsp68 *genes from other Diptera species (data not shown). These data enable us to suggest that *O. pardalina hsp68 *also represents a duplication of *hsp70 *gene. At the present time we can not discriminate between two hypotheses explaining the appearance of *hsp68*. The appearance of the *hsp68 *by duplication of *hsp70 *may occur very early in Diptera evolution and predate the split of Drosophilidae and Stratiomyidae. Alternatively *hsp68 *may appear independently in the evolution of *Drosophila *and Stratiomyidae and acquire similar functions and structure by convergence.

It is noteworthy that even for the genes located in inverted orientation, which apparently represents the primitive state of *hsp70 *in Diptera, the distance between the inverted copies in *O. pardalina *is four times as large as between the inverted copies in *S. singularior *(Figure 1A and 1B).

The different spacing of *hsp70 *genes in the species compared may resemble different abilities of their genome to efficiently respond to stress. The homogenized coding sequences and highly compact organization of *hsp70s *in *S. singularior *may be necessary for fast and efficient transcriptional response and thereby thermotolerance of the larvae to extreme and rapidly changing conditions of the environment.

On the other hand, *O. pardalina *inhabiting cold running waters with comparatively stable thermal conditions may not require such tight *hsp70 *cluster organization and, hence, underwent dispersal and pseudogenization of a few *hsp70 *copies in the process of evolution. Two *hsp70 *pseudogenes detected in the genome of this species in our studies may represent the remains of previously active genes. Previously we have shown that larvae of *O. pardalina *produce significantly less Hsp70 in response to heat shock in comparison with all other Stratiomyidae species investigated [8]. The observed drastic differences in the organization of *hsp70 *genes in *S. singularior *and *O. pardalina *may account for the differences in the thermotolerance and Hsp70 levels after HS treatment observed between these species.

Furthermore, HSE1 of *O. pardalina *is not canonical and contains only two 5 bp units. Therefore, the first canonical element (HSE2) in *O. pardalina *is located at significantly larger distance from the transcription start than in *S. singularior *(Figure 3). The differences in the number of canonical HSEs and their spacing may also contribute to different activity of *hsp70 *genes in the two species [33] and explain why *O. pardalina *exhibits lower thermotolerance in comparison with all other Stratiomyidae species studied so far [8].

It is also evident from Figure 3 that in the case of *O. pardalina*, the arrangement of promoter regions of all *hsp70 *genes is practically identical, while in the case of *S. singularior *the promoters of all members of *hsp70 *cluster differ significantly. These differences result from fine tuning of an *hsp70 *"battery" necessary for adaptation of the latter species to highly variable environmental conditions. Therefore, our results indicate that *O. pardalina hsp70 *genes may be locally adapted, whereas higher *hsp70 *polymorphism may have important evolutionary consequences for *S. singularior*.

The evolution of *hsp70 *genes in the species compared is quite different in many respects. The MK tests demonstrate that the divergence between individual *S. singularior hsp70 *genes is very low, especially considering the age of the duplications. This is consistent with concerted evolution in this species, and also strikingly different from the divergence between paralogs in *O. pardalina *(See Additional file 6: Table S1). In all cases, the number of shared polymorphisms between *S. singularior hsp70 *genes is highly unlikely to have occurred via parallel mutation (Table 1). The presence of multiple conversion-mediated shared SNPs and large conversion tracts in the alleles of the same *hsp70 *members support gene conversion as a mechanism of concerted evolution (and thus sharing polymorphism) among duplicated *hsp70 *genes of *S. singularior *species [34].

The overall pattern of conversion-mediated homogeneity among *hsp70 *genes in *S. singularior *that decays with distance from CDS, and polymorphic flanks that contain large indels, is consistent with previously described duplicated *hsp70s *in *Drosophila *[22]. We cannot exclude the occurrence of gene conversion in *O. pardalina*, because in this species we did not isolate multiple alleles of individual *hsp70 *genes. However, since the level of fixed differences between *hsp70 *genes in *O. pardalina *is several fold higher than in *S. singularior *(Additional file 6: Table S1), one may conclude that in the former species the conversion process is less efficient if present at all. The large distances between individual *hsp70 *genes in *O. pardalina *genome likely decrease the frequency of their conversion [35,36]. Whether the large distances also decrease their coordinate expression is an interesting question.

Several authors justly outlined many logical and statistical problems associated with using two-species comparison for studying adaptations [37], and we are well aware that unless similar correlation is obtained for other Stratiomyidae species or geographical populations, any generalizations are premature. However, a similar pattern of *hsp70 *organization has been previously described when studying two closely-related species of the *virilis *group of *Drosophila*, which replace one another along a latitudinal cline [14]. The less thermotolerant high-latitude species *D. lummei*, similar to *O. pardalina*, exhibits several-fold larger distance between tandemly arranged *hsp70 *copies in the cluster, in comparison with the closely-related more tolerant low-latitude *D. virilis *[14]. Interestingly, along these lines one of the *hsp70 *copies described in *D. lummei *was shown to be a nonfunctional pseudogene lacking the first 300 nucleotides of coding sequence. Similarly, two likely nonfunctional pseudogenes have been described in the present study in the less thermotolerant *O. pardalina*. Dispersed genomic arrangement is not absolutely required for *hsp70 *degeneration. The tight *hsp70 *clusters of the tropical island endemic *D. mauritiana *segregate multiple pseudogenes, although in this case a founder effect rather than relaxation of selection may modulate *hsp70 *pseudogene frequencies [22]. In spite of the approximately identical *hsp70 *copy number in Stratiomyidae and *Drosophila virilis *group species, there are several clear-cut differences in the organization and evolution of *hsp70 *cluster between these phyla. In species of the *virilis *group, not only the *hsp70 *transcriptional units but also the 3'-flanking sequences are highly conserved within *D. virilis *and between *D. lummei *and *D. virilis*. Thus, the 3'-flanking sequence of several *D. virilis hsp70 *genes is near identical for more than 1600 bp. Such an arrangement suggests tandem duplication of *hsp70 *copies by unequal crossing over in the course of *Drosophila *species evolution [13].

In contrast, our analysis detected only short, varying length regions of significant homology in the intergenic regions in *S. singularior hsp70 *cluster. Similarly, we detected only 650 bp of significant homology in the intergenic regions located at the vicinity of 5'-ends of *hsp70 *genes in *O. pardalina*.

At the present time, we can only speculate concerning the molecular mechanisms which may underlie the different arrangement of *hsp70 *genes in the species studied, although the pattern of compactness and conversion in *S. singularior vs. *dispersion and divergence in *O. pardalina *is clear. Several large deletions in *S. singularior*, including those spanning entire individual *hsp70 *genes, may be indicative of an evolutionary trend towards compactness to provide a highly coordinate response to the changing environmental conditions. The deletions may also be a consequence of conversion; the more homogenized the individual *hsp70 *units, the more likely they are also to participate in unequal crossover. On the other hand, the dispersed arrangement and interlocus divergence of *hsp70 *copies in *O. pardalina *may result from long-range recombination and relaxed selection on *hsp70 *sequences in a species living in very stable thermal conditions.

The analysis of *hsp70 *arrangement in other species and natural populations of Stratiomyidae and related taxa (work in progress) should help to further evaluate the evolution of *hsp70 *genes in the ecological context.

## Conclusions

The evolution of *hsp70 *genes in the species compared is quite different in many respects.

Although the *hsp70 *copy number does not differ significantly between less tolerant *O. pardalina *and more thermotolerant *S. singularior*, the general organization of *hsp70 *genes is divergent in these two species. While in *S. singularior *all *hsp70 *genes are confined to one genomic section of about 29 kb in length, in *O. pardalina *most of *hsp70 *genes are scattered in the genome and apparently located rather far from each if linked at all. However, in both species we have found two *hsp70 *copies in an inverted orientation, which represents a fundamental and probably ancient *hsp70 *gene unit. The divergence between individual *S. singularior hsp70 *genes is very low, especially considering the age of the duplications. This is consistent with concerted evolution in this species, and also strikingly different from the divergence between paralogs in *O. pardalina*. The observed drastic differences in the organization of *hsp70 *genes in *S. singularior *and *O. pardalina *may contribute to the differences in the thermotolerance between these species, and thus recapitulate the history of their thermal adaptation.

## Methods

### Insects and habitats

In this study, we have investigated two stratiomyids, *Stratiomys singularior *Harris, 1776 and *Oxycera pardalina *Meigen, 1822*. Stratiomys singularior *is a very common and widely distributed Transpalaearctic species [38] whose larvae develop in various shallow aquatic and semiaquatic habitats of standing waters. For this study, *S. singularoir *larvae were collected from the water margin zone of the highly mineralized chloride coastal Lake Koyashskoe in the Crimea. The measured temperature in the Crimean habitat of *S. singularior *in May-August varies from 15 to 30°C, but apparently, it is much lower in winter and can be significantly higher in summer. Water mineralization in the *S. singularior *habitat varied from 35 to 280 g/L, and could fluctuate around the highest values for weeks and months. *Oxycera pardalina *is also widely distributed in Western and Central Europe. The larvae of this species are confined to clear-water cold carbonate springs and streams [38]. The larvae were collected in the water margin zone of a spring near St. Petersburg characterized by a year-round stable low water temperature (5 - 10°C). The habitats of these populations and the techniques of sampling, identification and experiments have been described in detail in a previous paper [8].

### Preparation of RNA and cDNA synthesis

Total RNA from *S. singularior *larvae was prepared by the standard method with TRIZOL (Invitrogen). A cDNA library has been obtained from *S. singularior *total RNA using MINT cDNA kit (Evrogen) according to manufacturer's manual.

### Constructing and screening of cDNA library

Total double-stranded cDNA was cloned into the pAL-TA vector (Evrogen) using T4 DNA ligase (Promega). *E. coli *DH5α strain competent cells were transformed with ligation mix and inoculated onto LB agar Petri dishes. After overnight incubation at 32°C, colonies were transferred onto Hybond-N membranes (Amersham) and *in situ *hybridization was performed as described [39]. A full-size *hsp70 *gene of *D. melanogaster *was used as a labeled probe to screen cDNA library.

### 5'- and 3'-RACE analysis

The MINT cDNA kit was used for the first strand cDNA synthesis and amplification of 5'- and 3'-ends of cDNAs, as described by the manufacturer. For 5'- and 3'-end amplification outwards primers to 5'- and 3'-fragments of *S. singularior *and *O. pardalina hsp70 *coding region were used (See Additional file 15: Table S3 for primers used in the study). PCR-fragments obtained were cloned into pAL-TA vector (Evrogen) for sequencing.

### 2D electrophoresis and immunoblotting

Protein extraction and two-dimensional gel electrophoresis were performed as described by O'Farrell [40], with slight modifications [41]. In brief, lysates were prepared from larvae after exposure to various temperatures. After 2D electrophoresis of 50 μg of total protein, the proteins were transferred from gel into a nitrocellulose membrane (Hybond ECL; Amersham) according to the manufacturer's protocol. We used a monoclonal antibody specific to *D. melanogaster *inducible Hsp70 (antibody 7FB) and a monoclonal antibody which recognizes all members of *D. melanogaster *Hsp70 family (antibody 7.10.3) including inducible Hsp70, Hsp68 and HSC70 (the antibodies were kind gift of Dr. Susan Lindquist, Massachusetts Institute of Technology). Immune complexes were detected via chemoluminescence (ECL kit, Amersham) with appropriate peroxidase-conjugated anti-rat secondary antibodies (Millipore). Protein concentrations were normalized using BCA kit (Pierce) according to manufacturer's manual.

### Genomic libraries construction, screening and clone analyses

Genomic libraries from *S. singularior *and *O. pardalina *were prepared by partial *Sau3A *digestion of genomic DNA with subsequent ligation into the *BamHI *site of lambda Dash phage arms (Stratagene). Before ligation, restriction mixture was separated by ultracentrifugation in sucrose gradient for removal of short restriction fragments, and resulting fraction contained fragments with 14 - 23 kb length used for cloning. Gradient parameters were: 10 - 40% sucrose on 1 M NaCl, 20 mM Tris-HCl pH 8.0 and 5 mM EDTA with ultracentrifugation at 26 000 g during 24 h. Ligated DNA was packaged into phage particles using lambda packaging extracts (Stratagene). Recombinant phages were selected, amplified and screened using *E. coli *XL-Blue MRA (P2) host strains. For genomic libraries screening, *S. singularior hsp70 *full-length cDNA was used as a probe.

Isolated phages containing *hsp70 *genes were investigated by restriction analysis and hybridization, and subcloned into KS-Bluescript (pBluescript SK+) for subsequent sequencing.

### Southern blotting

Southern blot analysis of *S. singularior *and *O. pardalina *genomic DNA was performed as described [39]. Five micrograms of each DNA sample was digested with restriction endonucleases. After agarose gel electrophoresis, each gel was treated for 15 min in 0.25 M HCl and then incubated twice in denaturing buffer (1.5 M NaCl, 0.5 M NaOH) for 30 min. After 30 min incubation in neutralization buffer (1.5 M NaCl, 0.5 M Tris-HCl pH 7.5), gels were capillary-blotted onto nylon membranes and fixed by UV cross-linking using the UV Stratalinker 2400 (Stratagene) protocol. Standard high-stringency hybridization and wash conditions were used for Southern blot analysis. To detect *hsp70 *sequences in Southern blots and genomic libraries, different *EcoRI *fragments of cloned *S. singularior hsp70 *full-length cDNA (2340 nt in length) were labeled by random priming and used as probes.

The original screen of all libraries, individual recombinant clones analyses and Southern blots were performed using *S. singularior hsp70 *full-length cDNA, while Southern blots of *O. pardalina *genomic DNA were probed with 1250 - 2340 bp PCR-fragment of coding region of *O. pardalina hsp70 *gene.

### PCR conditions and primers

To obtain *S. singularior hsp70 *intergenic regions, we used primers outward from 5'- and 3'- *hsp70 *fragments. To obtain the *hsp70-hsp68 *intergenic region of *O. pardalina*, we used primers outward from the 3'-end of *hsp70 *and 5'-flanking fragment of *hsp68 *gene. To obtain the *O. pardalina *region located between *hsp70 *genes found in inverse orientation, we used primer outward from the 5'-end of *hsp70*. For amplification of the *O. pardalina hsp70 *coding region fragment used to probe Southern blots, inward primers for 5'- and 3'-ends of the gene were used (see Additional file 1, Table S3). PCR conditions depended on primer annealing time and temperature. All reactions contained 1.25 units of Encyclo DNA polymerase (Evrogen) per probe, 1.5 - 2.5 mM MgCl_2_, 0.2 mM of each dNTP, suitable quantity of DNA and 10 pM of each primer in 50 μl (total volume).

### Sequence analysis

Clones were sequenced with Sequenase (Amersham) and ABI 377 sequencers. Sequences were assembled manually and aligned using NCBI Blast and Vector NTI. Relevant sequence information has been deposited in GenBank. GenBank accessions numbers for *hsp70 *sequences are as following: *S. singularior *cDNA used as a probe for genomic libraries screen, EU523048; *S. singularior *genomic contigs assembled basing on several sequenced clones, HQ184404, HQ184405; *O. pardalina *genomic contigs, HM141535, HM141536, HM141537, HM141538, HM141539.

Whenever possible, *hsp70 *sequences were divided by eye into four segments: coding sequence ("CDS"), 5'- and 3'-untranslated regions ("UTRs"), and upstream promoter regions. The 5'-UTR was delineated by the TATA signal and last bases upstream of the start codon. The 3'-UTR was extended from the first bases downstream of the stop codon through the polyadenylation signal when possible. The promoter region was approximated as 400 - 600 bp upstream of the TATA signal.

Sequences were aligned using CLUSTALX [42], trimmed to eliminate end gaps, and unalignable sequences were removed (*i.e.*, sequences containing large indels or rearrangements, see below). BLAST (http://www.ncbi.nlm.nih.gov/BLAST) was utilized to assess identity of repetitive sequences around indels and rearrangements. Sequences were grouped across genes when they shared high homology (*e.g. *CDS, 5'-UTRs) or within genes when they diverged (*e.g. *most 3'-UTRs). After realignment of the trimmed, grouped sequences, custom perl scripts (available upon request) parsed alignments to generate figures and tally substitutions. For phylogenetic analysis, neighbor-joining tree building and 1000 bootstrap trials were conducted using CLUSTALX, MEGA [43] and Vector-NTI.

Analysis of polymorphism and divergence was conducted using DNASP [44]. Testing the probability of shared polymorphisms arising via gene conversion *vs. *independent mutation utilized the approximation of the hypergeometric distribution (implemented in DNASP; method described in [26]).

## List of abbreviations

Hsp70: heat shock protein 70.

## Authors' contributions

DG participated in the design of the study and genomic library analysis. IY participated in genomic libraries analysis and sequence alignments. BB has been involved in drafting of the manuscript and statistical analysis of the data. OZ participated in the design of the study and clones analysis. AP collected all biological material, assessed habitat conditions and determined the species, participated in drafting of the manuscript. ME participated in the design of the study, drafting of the manuscript and coordination of the whole project. All authors read and approved the final manuscript.

## Supplementary Material

Additional file 1 **Figure S1**. Alignment of *hsp70S1 *promoter sequences. Click here for file

Additional file 2 **Figure S2**. Alignment of *hsp70S2 *promoter sequences. Click here for file

Additional file 3 **Figure S3**. Alignment of *hsp70S3 *promoter sequences. Click here for file

Additional file 4 **Figure S4**. Alignment of *hsp70S4 *promoter sequences. Click here for file

Additional file 5 **Figure S5**. Alignment of *hsp70S5 *promoter sequences. Click here for file

Additional file 6 **Table S1**. Numbers of silent and replacement fixed differences between *O. pardalina hsp70 *genes. Click here for file

Additional file 7 **Table S2**. Results of MK (McDonald-Kreitman) tests. Click here for file

Additional file 8 **Figure S6**. Alignment of *Stratiomys hsp70 *coding sequences. Click here for file

Additional file 9 **Figure S7**. Alignment of *Stratiomys hsp70 *5'-UTR sequences. Click here for file

Additional file 10 **Figure S8**. Alignment of *hsp70S1 *3'-UTR sequences. Click here for file

Additional file 11 **Figure S9**. Alignment of *hsp70S2 *and *hsp70S3 *3'-UTR sequences. Click here for file

Additional file 12 **Figure S10**. Alignment of *hsp70S4 *3'-UTR sequences. Click here for file

Additional file 13 **Figure S11**. Alignment of *hsp70S5 *3'-UTR sequences. Click here for file

Additional file 14 **Figure S12. Deduced amino acids sequence of Hsp70S1 (*S. singularior*), Hsp70P1 and Hsp68 (both from *O. pardalina*)**. Comparison with paralogs from several organisms exhibiting homology to Stratiomyidae Hsp70. Click here for file

Additional file 15 **Table S3**: Primers for PCR and RACEs, used in the study. Click here for file
