# Supplementary material for: Organization and evolution of hsp70 clusters strikingly differ in two species of Stratiomyidae (Diptera) inhabiting thermally contrasting environments
Source: BMC Evol Biol. 2011 Mar 22;11:74. doi: 10.1186/1471-2148-11-74 (PMC3071340; doi:10.1186/1471-2148-11-74)
Supplement: Additional file 1 — Figure S1. Alignment of hsp70S1 promoter sequences. [file 1471-2148-11-74-S1.DOC]

**Additional file 1: Figure S1. Alignment of *hsp70S1* promoter sequences.** Sequences end at last nucleotide before TATA box. Alleles named by phage number (superscript). Dots indicated identical nucleotides, dashes are gaps. Consensus heat shock elements (HSEs) in green; likely HSEs in yellow.

*hsp70S1^10^* AATGTGAGTTATAAGATACTTTTTCCTCATTTTCCACATTATTTCTATTGCACATTACTT

*hsp70S1^71^* .............................................C..............

*hsp70S1^52^* .............................................C..............

*hsp70S1^10^* AAAACTTAATCGAAAATCTTTGCGTTTCCGATTATGTTTTCATTCATGAAAATATTCAAG

*hsp70S1^71^* ............................................................

*hsp70S1^52^* ............................................................

*hsp70S1^10^* ACTCATCGATAGGAATAGCAATGACGTATGAGTACAATTTACCCAACATGGTCACTTCCT

*hsp70S1^71^* .......................................A....................

*hsp70S1^52^* .......................................A....................

*hsp70S1^10^* GTCAATCTAAGAACATTTATCTGCAATCAATTGATGTTCGTCGCATTTATATTTTTATCT

*hsp70S1^71^* ............................................................

*hsp70S1^52^* ............................................................

*hsp70S1^10^* ACTCAATTATAAACAAATGCAACTTAGGCAGTCATCGACGTGCCATTTCCATTGAAGCAC

*hsp70S1^71^* ....................G.......................................

*hsp70S1^52^* ....................G.......................................

*hsp70S1^10^* CATCCATAATAAATTGACCTATTTGCGTAATTTGTTGTTTTGCTATTAACGCTATCTGAC

*hsp70S1^71^* .....................C.........G...................T........

*hsp70S1^52^* .....................C.........G...................T........

*hsp70S1^10^* TCAAACCGAGCGAACATTCCAGTAACATACAGAAAAATCTCAAACTTTCCACTGCTGAAA

*hsp70S1^71^* ............................................................

*hsp70S1^52^* ...............................................-............

*hsp70S1^10^* GTATCTT--AAACAAAATCGATGCCGACAATCGTTCAAAGCTTCTCGATACATCTACAGA

*hsp70S1^71^* ............................................................

*hsp70S1^52^* .......TA...................................................

*hsp70S1^10^* GTTCCAACAGAATGTTCCCGACGATTTACCAGGAG

*hsp70S1^71^* ...................................

*hsp70S1^52^* ...................................
