# Supplementary material for: Organization and evolution of hsp70 clusters strikingly differ in two species of Stratiomyidae (Diptera) inhabiting thermally contrasting environments
Source: BMC Evol Biol. 2011 Mar 22;11:74. doi: 10.1186/1471-2148-11-74 (PMC3071340; doi:10.1186/1471-2148-11-74)
Supplement: Additional file 2 — Figure S2. Alignment of hsp70S2 promoter sequences. [file 1471-2148-11-74-S2.DOC]

**Additional file 2: Figure S2. Alignment of *hsp70S2* promoter sequences.** Sequences end at last nucleotide before TATA box. Alleles named by phage number (superscript). Dots indicated identical nucleotides, dashes are gaps. Consensus heat shock elements (HSEs) in green; likely HSEs in yellow.

*hsp70S2^10^* GTATTGACCAATTTAAAAGAGAATATAAAAAGCCAAACTGAAAACGTTGGGAATACACGT

*hsp70S2^71^* .................-........G...G---C...............-........G

*hsp70S2^10^* GATGTTCTATTCATTATGTTTAATAAGAACGCATTCAGAAGTCTTTTAACGTTTTCTCAA

*hsp70S2^71^* .........................................................T..

*hsp70S2^10^* AGGCCTCCGCAGTTTCGATTGACTGCGTTTGCTACCTACAATTAAAGCATGATAGCTAGG

*hsp70S2^71^* ............................................................

*hsp70S2^10^* TGACAAAGAATATGAATGTAAATCAGCAATAATGGGTAATCGCACTTTATTTA---GGGT

*hsp70S2^71^* .........................T...........................TTTA..G

*hsp70S2^10^* CTTCCATGAAATGACGCATTCTCAGTGGAA-TTTTAATAAACTCTCGATATTTCAAGCAT

*hsp70S2^71^* TC.T..................G.......A....................C........

*hsp70S2^10^* ATATCAGATCGAA-CTGCTTATTATTAATTCAACACGACTCACTACTGCATAATCGATAA

*hsp70S2^71^* .............A.........................................A....

*hsp70S2^10^* CCTCCATAAAAGATTTTGGAGGATTGAAGCACACACGTCTTTATTCTTTCCTAAACTTTT

*hsp70S2^71^* .AC...............A..A.......T........A..................C..

*hsp70S2^10^* GCATCATT-CCATTCATATACGTACAACCTCAATTGAAAATTTTCTAGAGGTTTCCTAAT

*hsp70S2^71^* ........T...............A..........C........................

*hsp70S2^10^* GTGTTTTCATTTATCGGAAATTTCGAGATACATCCACAGAGTTCTAACAGAATGTTCCCG

*hsp70S2^71^* ...........C......................T.........C...............

*hsp70S2^10^* ACGATTTACCAGGAG

*hsp70S2^71^* ............A..
