# Supplementary material for: Organization and evolution of hsp70 clusters strikingly differ in two species of Stratiomyidae (Diptera) inhabiting thermally contrasting environments
Source: BMC Evol Biol. 2011 Mar 22;11:74. doi: 10.1186/1471-2148-11-74 (PMC3071340; doi:10.1186/1471-2148-11-74)
Supplement: Additional file 3 — Figure S3. Alignment of hsp70S3 promoter sequences. [file 1471-2148-11-74-S3.DOC]

**Additional file 3: Figure S3. Alignment of *hsp70S3* promoter sequences.** Sequences end at last nucleotide before TATA box. Alleles named by phage number (superscript). Dots indicated identical nucleotides, dashes are gaps. Consensus heat shock elements (HSEs) in green; likely HSEs in yellow. Triangle marks insertion of unaligned repetitive sequence in *hsp70S3^52^*.

*hsp70S3^10^* ATACCAAAGGAAAATAATTTTCTCACAAACTTTTCACTTAAATGTGCAAATTATTTGCTT

*hsp70S3^33^* ....................G.......................................

*hsp70S3^10^* TTTCTGCAAATCGTCGATCCTTTATTTGTATTACAATCGCATCTCACGCTTCCAATTAAT

*hsp70S3^33^* ............................................................

*hsp70S3^10^* TCAAATACTTATTATTAACAGCCTTTCTAAACCGCTGGTGTTACTTGTTTCATTTTTCCT

*hsp70S3^33^* ............................................................

*hsp70S3^10^* CCGTCTGCTTGGGTGCTCATTAGTCAGATAATACGTC-ATCGACTCTCCACGAATCTTAA

*hsp70S3^33^* .........................................................-..

*hsp70S3^51^* .G...........................C...............-......

*hsp70S3^10^* AAAACATTAAAAACAATTTTAAATTAACAACACATTAGTCAGAAGAAGACGCATTCGCAA

*hsp70S3^33^* ............................................................

*hsp70S3^51^* ............................................................

*hsp70S3^10^* CAATAGCTAGATAATGGATCGACATCATTGATTCGAAGATTCCCGTATTTGCCAGAACAA

*hsp70S3^33^* ............................................................

*hsp70S3^51^* ............................................................

*hsp70S3^10^* TCCCAAACATCCAACCCTTATGTATCATATGGAGTATTCAAAACACTCAACTCCTATTCC

*hsp70S3^33^* ............................................................

*hsp70S3^51^* ............................................................

TE remnants

*hsp70S3^52^*

*hsp70S3^10^* ACTTCAACAATTTTCGAAGTCTCGACTCTTCTC CAGAGTT

*hsp70S3^33^* ....G............................ .......

*hsp70S3^51^* ................................. .......

*hsp70S3^52^* .......

*hsp70S3^10^* CCAACAGAATGTTCCCGACGATTTACCAGGAG

*hsp70S3^33^* ................................

*hsp70S3^51^* ................................

*hsp70S3^52^* .............................A..
