# Supplementary material for: Organization and evolution of hsp70 clusters strikingly differ in two species of Stratiomyidae (Diptera) inhabiting thermally contrasting environments
Source: BMC Evol Biol. 2011 Mar 22;11:74. doi: 10.1186/1471-2148-11-74 (PMC3071340; doi:10.1186/1471-2148-11-74)
Supplement: Additional file 4 — Figure S4. Alignment of hsp70S4 promoter sequences. [file 1471-2148-11-74-S4.DOC]

**Additional file 4: Figure S4.** **Alignment of *hsp70S4* promoter sequences.** Sequences end at last nucleotide before TATA box. Alleles named by phage number (superscript). Dots indicated identical nucleotides, dashes are gaps. Consensus heat shock elements (HSEs) in green.

*hsp70S4^5^* ATCTGCAAATTAAACTTTAGTGCCAATAAAATTATGGCTTTAAAGTATTGTATTTGAATC

*hsp70S4^17^* ...................................................C........

*hsp70S4^52^* ............................................................

*hsp70S4^5^* AAAAAGATTCATTCTGTATTCAAATATATCTCACATGTGTATAGCGGCTCCTAATACTTT

*hsp70S4^17^* ............................................................

*hsp70S4^52^* ........................................................T...

*hsp70S4^5^* AAAATACATCAGTTAACATCAGCAAACTAGCATTAATGCTACAGGGCAGTTTACGCTACA

*hsp70S4^17^* ............................................................

*hsp70S4^52^* ............................................................

*hsp70S4^5^* ATTTCACTCTTTTTCATTATGAATACATTCATAAGCTTTAGATCACTAGTTATCACACAC

*hsp70S4^17^* ............................A...............................

*hsp70S4^52^* ................................................A...........

*hsp70S4^5^* TAGCTTTCAACAACTAAACAGTCATGCTTGCATTTAAAGATTTGTTATTTTT-ACTCGTT

*hsp70S4^17^* ....................................................T.......

*hsp70S4^52^* ............................................................

*hsp70S4^5^* TAAAGATAAACAAATGTCGTCGACACGGTCGTCCACTTCCCAATCTTTT-CATTACACCA

*hsp70S4^17^* -................................................T..........

*hsp70S4^52^* ......................................G.....................

*hsp70S4^5^* ATCAACGTATGTTTGTTTTGGTAATCATGCTATATGACTCACTGCGAATGAACATTCCAG

*hsp70S4^17^* .........................................A..................

*hsp70S4^52^* .........................................A..................

*hsp70S4^5^* TAACATAAAGAAATATCTCAAACTTTCCAATGCTGAAAGTATCTTAAACAAAATCGATGC

*hsp70S4^17^* ............................................................

*hsp70S4^52^* ..........................................T.................

*hsp70S4^5^* CGACAAACGTTCGAAGCTTCTCGATACATCTACAGAGTTCCAACAGAATGTTCCCGACGA

*hsp70S4^17^* ............................................................

*hsp70S4^52^* ........................................T...................

*hsp70S4^5^* TTTACCAGAAG

*hsp70S4^17^* ...........

*hsp70S4^52^* ........G..
