# Supplementary material for: Organization and evolution of hsp70 clusters strikingly differ in two species of Stratiomyidae (Diptera) inhabiting thermally contrasting environments
Source: BMC Evol Biol. 2011 Mar 22;11:74. doi: 10.1186/1471-2148-11-74 (PMC3071340; doi:10.1186/1471-2148-11-74)
Supplement: Additional file 5 — Figure S5. Alignment of hsp70S5 promoter sequences. [file 1471-2148-11-74-S5.DOC]

**Additional file 5: Figure S5.** **Alignment of *hsp70S5* promoter sequences.** Sequences end at last nucleotide before TATA box. Alleles named by phage number (superscript). Dots indicated identical nucleotides, dashes are gaps. Consensus heat shock elements (HSEs) in green. Triangle marks insertion of unaligned repetitive sequence in *hsp70S5^33^*. Grey bar marks *hsp70S4* derived conversion tract.

*hsp70S5^17^* TAACCAACGTAAAATGACTTTTGTTAAAATATTTTTCTAAAAGATGTTTTATAATAATTC

*hsp70S5^63^* -...........................................................

*hsp70S5^8^* ............................................................

*hsp70S5^17^* CAATTACCGCGAAATTTTGAATCGGAGTAGTAATATTGCGGTTCGTGAAATGTGTTTGGG

*hsp70S5^63^* ..........A..................T..............................

*hsp70S5^8^* ..........A..................T..............................

*hsp70S5^17^* TATTTGATCGTTCCACTAAATGTAGATGAATGCAACTAACGTTCAGTTCTTAATTTGAAC

*hsp70S5^63^* .............T..........C...................................

*hsp70S5^8^* .............T..........C...................................

*hsp70S5^17^* TACTCGGAATACATTT-GGTGATTGCCTACCAATCGCAAAATATGCAAAATCGCTCGAAC

*hsp70S5^63^* ....T...........T...........................................

*hsp70S5^8^* ....T.......................................................

*hsp70S5^17^* TCTTTCTTGGCAAAACTATAAACGTAATCTCTCTAACGACCGATTGCATAATTCCACTCC

*hsp70S5^63^* ......A.....G...............................................

*hsp70S5^8^* ......A.....G...............................................

*hsp70S5^17^* ATTCTAACAAATAACAAATGCCCTAATTTCACATTCATCGAAAATATGACTCAAAATCAT

*hsp70S5^63^* .................T..........................................

*hsp70S5^8^* .................T..........................................

*hsp70S5^33^* -...........-

*hsp70S5^17^* TCGCCCAAACATAATAAACATCATGCCAACCCGAATCGAATATTCTAGCAGCAGCCAGAA

*hsp70S5^63^* .....................AT.....................................

*hsp70S5^8^* .....................AT.....................................

*hsp70S5^33^*

*hsp70S5^17^* ACACATCGTTTTTCCGCTGCTGTGTAACATGATGGACACACTCACCTCATCCGAAACTAT

*hsp70S5^63^* .T.T.................T......................................

*hsp70S5^8^* .T.T.................T......................................

*hsp70S5^33^*

TE remnants

*hsp70S5^33^*

*hsp70S5^17^* TTTCGTAGATA CGAAATTTCTCGACTAGTCTACAGAGTTC

*hsp70S5^63^* ........... .............................

*hsp70S5^8^* ........... .............................

*hsp70S5^33^*  *....*GC.......TACA............

*hsp70S5^17^* CAACAGAATGTTCCCGATGATTTACCAGAAG

*hsp70S5^63^* ...............................

*hsp70S5^8^* ...............................

*hsp70S5^33^* T........T.......C..........G..
