# Supplementary material for: Organization and evolution of hsp70 clusters strikingly differ in two species of Stratiomyidae (Diptera) inhabiting thermally contrasting environments
Source: BMC Evol Biol. 2011 Mar 22;11:74. doi: 10.1186/1471-2148-11-74 (PMC3071340; doi:10.1186/1471-2148-11-74)
Supplement: Additional file 6 — Table S1. Numbers of silent and replacement fixed differences between O. pardalina hsp70 genes. [file 1471-2148-11-74-S6.DOC]

**Additional file 6: Table S1** Numbers of silent and replacement fixed differences between *O. pardalina hsp70* genes. Results of quantitative comparison of silent and replacement differences when four individual *hsp70* copies of *O.pardalina* were analyzed.

|  | P3 | | P4 | |
| --- | --- | --- | --- | --- |
|  | Silent | Replacement | Silent | Replacement |
| P1 | 27 | 5 | 25 | 10 |
| P3 |  |  | 20 | 7 |
