# Supplementary material for: Organization and evolution of hsp70 clusters strikingly differ in two species of Stratiomyidae (Diptera) inhabiting thermally contrasting environments
Source: BMC Evol Biol. 2011 Mar 22;11:74. doi: 10.1186/1471-2148-11-74 (PMC3071340; doi:10.1186/1471-2148-11-74)
Supplement: Additional file 7 — Table S2. Results of MK (McDonald-Kreitman) tests. [file 1471-2148-11-74-S7.DOC]

**Additional file 7:** **Table S2. Results of MK (McDonald-Kreitman) tests.**

A)

| Gene |  | P1 | | P3 | | P4 | |
| --- | --- | --- | --- | --- | --- | --- | --- |
|  |  | Fixed | Poly | Fixed | Poly | Fixed | Poly |
| S1 | Sil | 309 | 24 | 311 | 24 | 311 | 24 |
|  | Rep | 49 | 6 | 45 | 6 | 50 | 6 |
| S2 | Sil | 314 | 17 | 315 | 17 | 315 | 51 |
|  | Rep | 50 | 2 | 46 | 2 | 17 | 2 |
| S3 | Sil | 314 | 18 | 316 | 18 | 316 | 18 |
|  | Rep | 48 | 9 | 44 | 9 | 49 | 9 |
| S4 | Sil | 316 | 29 | 320 | 29 | 319 | 29 |
|  | Rep | 49 | 7 | 45 | 7 | 50 | 7 |
| S5 | Sil | 306 | 53 | 309 | 53 | 308 | 53 |
|  | Rep | 47 | 10 | 43 | 10 | 48 | 10 |

B)

| Gene |  | S2 | | S3 | | S4 | | S5 | |
| --- | --- | --- | --- | --- | --- | --- | --- | --- | --- |
|  |  | Fixed | Poly | Fixed | Poly | Fixed | Poly | Fixed | Poly |
| S1 | Sil | 3 | 34 | 3 | 32 | 8 | 41 | 4 | 64 |
|  | Rep | 2 | 8 | 1 | 11 | 1 | 10 | 1 | 15 |
| S2 | Sil |  |  | 2 | 28 | 4 | 41 | 8 | 62 |
|  | Rep |  |  | 2 | 10 | 2 | 9 | 3 | 12 |
| S3 | Sil |  |  |  |  | 6 | 40 | 6 | 62 |
|  | Rep |  |  |  |  | 2 | 14 | 2 | 17 |
| S4 | Sil |  |  |  |  |  |  | 9 | 65 |
|  | Rep |  |  |  |  |  |  | 1 | 17 |

Numbers of silent and replacement polymorphisms and differences fixed between *S. singularior* and *O. pardalina hsp70* genes (A), and between *S. singularior hsp70* genes (B). Sil, number of silent substitutions. Rep, number of replacement substitutions. Fixed, number of substitutions fixed between genes. Poly, number of polymorphic substitutions within genes. Color indicates significance level of G test of independence (grey, *p* < 0.05; black, *p* < 0.01).
