# Supplementary material for: Organization and evolution of hsp70 clusters strikingly differ in two species of Stratiomyidae (Diptera) inhabiting thermally contrasting environments
Source: BMC Evol Biol. 2011 Mar 22;11:74. doi: 10.1186/1471-2148-11-74 (PMC3071340; doi:10.1186/1471-2148-11-74)
Supplement: Additional file 8 — Figure S6. Alignment of Stratiomys hsp70 coding sequences. [file 1471-2148-11-74-S8.DOC]

A**dditional file 8: Figure S6. Alignment of *Stratiomys hsp70* coding sequences.** Sequences begin on start and end on stop codon. Alleles named by phage number (superscript). Dots indicated identical nucleotides. Black bars indicate conversion-mediated shared SNPs.

*hsp70S1^10^* ATGCCTGCAGTAGGAATTGATTTGGGAACAACATTCTCGTGCGTTGGAGTATTCCAGCAC

*hsp70S1^52^* ............................................................

*hsp70S1^71^* ............................................................

*hsp70S2^10^* ............................................................

*hsp70S2^71^* .........................................T..................

*hsp70S3^10^* ............................................................

*hsp70S3^33^* ............................................................

*hsp70S3^51^* ............................................................

*hsp70S3^52^* .........................................T..................

*hsp70S4^17^* .........................................T..................

*hsp70S4^52^* .........................................T..................

*hsp70S5^8^* .........................................T..................

*hsp70S5^17^* ............................................................

*hsp70S5^33^* ............................................................

*hsp70S5^51^* ............................................................

*hsp70S5^63^* ............................................................

*hsp70S1^10^* GGGAAAGTCGAAATCATCGCCAACGACCAAGGAAACAGAACAACTCCCAGCTATGTGGCG

*hsp70S1^52^* ............................................................

*hsp70S1^71^* ............................................................

*hsp70S2^10^* ....................T.......................................

*hsp70S2^71^* ............................................................

*hsp70S3^10^* ............................................................

*hsp70S3^33^* ............................................................

*hsp70S3^51^* ............................................................

*hsp70S3^52^* ............................................................

*hsp70S4^17^* ....................T.......................................

*hsp70S4^52^* ....................T...........G...........................

*hsp70S5^8^* ....................T...........G.......................T...

*hsp70S5^17^* ............................................................

*hsp70S5^33^* ............................................................

*hsp70S5^51^* ............................................................

*hsp70S5^63^* ................................G.......................T...

*hsp70S1^10^* TTCACGGACTCAGAACGCCTGATAGGCGATGCAGCCAAGAACCAGGTGGCTATGAACCCG

*hsp70S1^52^* ...........G................................................

*hsp70S1^71^* ............................................................

*hsp70S2^10^* ............................................................

*hsp70S2^71^* ............................................................

*hsp70S3^10^* ............................................................

*hsp70S3^33^* ............................................................

*hsp70S3^51^* ............................................................

*hsp70S3^52^* ...........G................................................

*hsp70S4^17^* ............................................................

*hsp70S4^52^* ............................................................

*hsp70S5^8^* ............................................................

*hsp70S5^17^* ............................................................

*hsp70S5^33^* ............................................................

*hsp70S5^51^* ............................................................

*hsp70S5^63^* ............................................................

*hsp70S1^10^* AAGAACACTGTTTTCGACGCGAAACGGTTGATTGGACGGAAATATGACGATCCGAAGATT

*hsp70S1^52^* ..................................................C.........

*hsp70S1^71^* ..................................................C.........

*hsp70S2^10^* ............................................................

*hsp70S2^71^* ..................................................C.........

*hsp70S3^10^* ..................................................C.........

*hsp70S3^33^* ..................................................C.........

*hsp70S3^51^* ...................................C..............C.........

*hsp70S3^52^* ..A...............................................C.........

*hsp70S4^17^* ............................................................

*hsp70S4^52^* .....T......................................................

*hsp70S5^8^* ..................................................C.........

*hsp70S5^17^* ..................................................C.........

*hsp70S5^33^* ..................................................C.........

*hsp70S5^51^* ..................................................C.........

*hsp70S5^63^* ..................................................C.........

*hsp70S1^10^* CAGGAGGACTTGAGGCATTGGCCATTCACCGTGAAGAGCGACAGTGGAAAGCCGAAAATC

*hsp70S1^52^* ............................................................

*hsp70S1^71^* ............................................................

*hsp70S2^10^* ...............A................A...........................

*hsp70S2^71^* ...............A............................................

*hsp70S3^10^* ............................................................

*hsp70S3^33^* ...............A............................................

*hsp70S3^51^* ............................................................

*hsp70S3^52^* ............................................................

*hsp70S4^17^* ................................A...........................

*hsp70S4^52^* ................................A...........................

*hsp70S5^8^* ...............A...........................T................

*hsp70S5^17^* ...............A............................................

*hsp70S5^33^* ............................................................

*hsp70S5^51^* ............................................................

*hsp70S5^63^* ...............A...........................T................

*hsp70S1^10^* TGCGTGGAATTCAAAGGCGAGCAGAAACGCTTCGCGCCCGAGGAAATCAGTTCAATGGTG

*hsp70S1^52^* ......................................T.....................

*hsp70S1^71^* ......................................T.....................

*hsp70S2^10^* ............................................................

*hsp70S2^71^* ......................................T.....................

*hsp70S3^10^* ......................................T.....................

*hsp70S3^33^* ......................................T.....................

*hsp70S3^51^* ......................................T.....................

*hsp70S3^52^* ......................................T.....................

*hsp70S4^17^* ............................................................

*hsp70S4^52^* ......................................T..................T..

*hsp70S5^8^* .................T....................T.....................

*hsp70S5^17^* ............................................................

*hsp70S5^33^* ......................................T.....................

*hsp70S5^51^* ......................................T.....................

*hsp70S5^63^* .................T....................T.....................

*hsp70S1^10^* TTGACGAAGATGAAGGAAACGGCGGAAGCGTACTTGGGAGAATCGGTTACAGACGCAGTG

*hsp70S1^52^* ............................................................

*hsp70S1^71^* ............................................................

*hsp70S2^10^* ............................................................

*hsp70S2^71^* ............................................................

*hsp70S3^10^* ............................................................

*hsp70S3^33^* ............................................................

*hsp70S3^51^* ............................................................

*hsp70S3^52^* ............................................................

*hsp70S4^17^* ............................................................

*hsp70S4^52^* ...................................................A.......A

*hsp70S5^8^* ...........................................................A

*hsp70S5^17^* .............................T..............A...............

*hsp70S5^33^* ............................................................

*hsp70S5^51^* ............................................................

*hsp70S5^63^* ...........................................................A

*hsp70S1^10^* ATCACAGTTCCAGCATATTTCAATGACTCGCAGCGTCAAGCGACGAAGGATGCTGGAGCG

*hsp70S1^52^* ............................................................

*hsp70S1^71^* ............................................................

*hsp70S2^10^* ............................................................

*hsp70S2^71^* ............................................................

*hsp70S3^10^* ............................................................

*hsp70S3^33^* ............................................................

*hsp70S3^51^* ............................................................

*hsp70S3^52^* ............................................................

*hsp70S4^17^* ............................................................

*hsp70S4^52^* .....T..A...................................................

*hsp70S5^8^* ........A...................................................

*hsp70S5^17^* ................................A...........................

*hsp70S5^33^* ............................................................

*hsp70S5^51^* ............................................................

*hsp70S5^63^* ........A...................................................

*hsp70S1^10^* ATCGCTGGTTTGAATGTGCTGAGGATTATCAACGAACCTACAGCAGCAGCGCTGGCCTAT

*hsp70S1^52^* ......................................G........T............

*hsp70S1^71^* ......................................G........T............

*hsp70S2^10^* ......................................G........T............

*hsp70S2^71^* ......A...............................G........T............

*hsp70S3^10^* ......................................G........T............

*hsp70S3^33^* ............................................................

*hsp70S3^51^* ......................................G........T............

*hsp70S3^52^* ......A...............................G........T............

*hsp70S4^17^* ......................................G........T............

*hsp70S4^52^* ......................................G........T............

*hsp70S5^8^* ...............................................T............

*hsp70S5^17^* ...............................................T............

*hsp70S5^33^* ......................................G........T............

*hsp70S5^51^* ......................................G........T............

*hsp70S5^63^* ...............................................T............

*hsp70S1^10^* GGCCTGGACAAGAATCTCAAAGGTGAACGCAACGTGTTGATCTTCGACTTGGGTGGTGGC

*hsp70S1^52^* ......................................A.....................

*hsp70S1^71^* ......................................A.....................

*hsp70S2^10^* ......................................A.....................

*hsp70S2^71^* ......................................A.....................

*hsp70S3^10^* ......................................A.....................

*hsp70S3^33^* ......................................A.....................

*hsp70S3^51^* ......................................A.....................

*hsp70S3^52^* ......................................A.....................

*hsp70S4^17^* ............................................................

*hsp70S4^52^* .....A................................A.....T...............

*hsp70S5^8^* ........T...................................................

*hsp70S5^17^* .....A......................................................

*hsp70S5^33^* ........T............................................C......

*hsp70S5^51^* ........T............................................C......

*hsp70S5^63^* ........T...................................................

*hsp70S1^10^* ACATTCGATGTTTCGATCCTGACGATCGACGAAGGATCATTGTTCGAAGTACGAGCGACG

*hsp70S1^52^* ..................T.........................................

*hsp70S1^71^* ..................T.........................................

*hsp70S2^10^* ............................................................

*hsp70S2^71^* ..................T.........................................

*hsp70S3^10^* ............................................................

*hsp70S3^33^* ..........................A.................................

*hsp70S3^51^* ............................................................

*hsp70S3^52^* ..................T.........................................

*hsp70S4^17^* ............................................................

*hsp70S4^52^* ..........................A.................T...............

*hsp70S5^8^* .....................................................G......

*hsp70S5^17^* ..........................A.................T...............

*hsp70S5^33^* ....................A.....A.................T...............

*hsp70S5^51^* ....................A.....A.................T...............

*hsp70S5^63^* .....................................................G......

*hsp70S1^10^* GCCGGTGATACTCACCTGGGAGGTGAGGACTTCGATAACCGCTTGGTGTCGTATTTGGCT

*hsp70S1^52^* .....C..C..C..T.....T.......................................

*hsp70S1^71^* .....C..C..C..T.....T.......................................

*hsp70S2^10^* .....C..C...................................................

*hsp70S2^71^* .....C..C..C..T.....T.......................................

*hsp70S3^10^* .....C..C..C..T.....T.......................................

*hsp70S3^33^* .....C..C...................................................

*hsp70S3^51^* .....C..C..C..T.....T.......................................

*hsp70S3^52^* .....C..C..C..T.....T.......................................

*hsp70S4^17^* .....C..C...................................................

*hsp70S4^52^* .....C..C..C..T.....T..............................C........

*hsp70S5^8^* ...................................................C........

*hsp70S5^17^* .....C..C.....T.....T....................T........AC........

*hsp70S5^33^* .....C..C.....T..........................T.........C........

*hsp70S5^51^* .....C..C.....T..........................T.....A...C........

*hsp70S5^63^* ...................................................C........

*hsp70S1^10^* GATGAGTTCAAACGCAAGTATAAGAAGGACCTGAGATCGAATCCACGAGCACTGAGACGC

*hsp70S1^52^* ............................................................

*hsp70S1^71^* ............................................................

*hsp70S2^10^* ............................................................

*hsp70S2^71^* ............................................................

*hsp70S3^10^* ............................................................

*hsp70S3^33^* ............................................................

*hsp70S3^51^* ............................................................

*hsp70S3^52^* ............................................................

*hsp70S4^17^* ............................................................

*hsp70S4^52^* ............................................................

*hsp70S5^8^* ................................C...........................

*hsp70S5^17^* .............A..................A...........................

*hsp70S5^33^* ........G.......................A...........................

*hsp70S5^51^* ................................A...........................

*hsp70S5^63^* ................................C...........................

*hsp70S1^10^* TTGAGGACGGCGGCTGAACGAGCCAAACGAACATTATCATCGAGCACTGAAACCACCATC

*hsp70S1^52^* ........................................................G...

*hsp70S1^71^* ........................................................G...

*hsp70S2^10^* ..............G.................T..........................T

*hsp70S2^71^* ............................................................

*hsp70S3^10^* ..............G.................T...........................

*hsp70S3^33^* ..............G.................T...........................

*hsp70S3^51^* ..............G.................T...........................

*hsp70S3^52^* ............................................................

*hsp70S4^17^* ................................T...........................

*hsp70S4^52^* ........................................................G...

*hsp70S5^8^* ..............G.........................................G...

*hsp70S5^17^* ........................................................G...

*hsp70S5^33^* ........................................................G...

*hsp70S5^51^* ............................................................

*hsp70S5^63^* ..............G.........................................G...

*hsp70S1^10^* GAGATTGACGCTTTGTACGAAGGAGTTGACTTCTACACGAAAGTGTCAAGAGCTCGATTT

*hsp70S1^52^* ............................................................

*hsp70S1^71^* ............................................................

*hsp70S2^10^* ............................................................

*hsp70S2^71^* ............................................................

*hsp70S3^10^* ............................................................

*hsp70S3^33^* ................G...........................................

*hsp70S3^51^* ............................................................

*hsp70S3^52^* ............................................................

*hsp70S4^17^* ............................................................

*hsp70S4^52^* ............................................................

*hsp70S5^8^* ............................................................

*hsp70S5^17^* ............................................................

*hsp70S5^33^* .................................C..........................

*hsp70S5^51^* .................................C..........................

*hsp70S5^63^* ............................................................

*hsp70S1^10^* GAGGAACTGTGTGCGGACTTGTTCCGATCCACATTGCAGCCAGTGGAGAAGGCTTTGAAT

*hsp70S1^52^* .........A...........................................C......

*hsp70S1^71^* .........A...........................................C......

*hsp70S2^10^* ............................................................

*hsp70S2^71^* ............................................................

*hsp70S3^10^* ............................................................

*hsp70S3^33^* ............................................................

*hsp70S3^51^* ............................................................

*hsp70S3^52^* ............................................................

*hsp70S4^17^* ............................................................

*hsp70S4^52^* .........A...........................................C......

*hsp70S5^8^* ...............A..........G.............................A...

*hsp70S5^17^* .....................................................C......

*hsp70S5^33^* ............................................................

*hsp70S5^51^* ............................................................

*hsp70S5^63^* ...............A..........G.............................A...

*hsp70S1^10^* GACGCGAAAATGGACAAGTCGCAGATCCATGACATCGTGATGGTAGGCGGCTCGACTCGA

*hsp70S1^52^* ........G...................................................

*hsp70S1^71^* ........G...................................................

*hsp70S2^10^* ............................................................

*hsp70S2^71^* ............................................................

*hsp70S3^10^* ............................................................

*hsp70S3^33^* ............................................................

*hsp70S3^51^* ............................................................

*hsp70S3^52^* ........G...................................................

*hsp70S4^17^* ........G...................................................

*hsp70S4^52^* ........G...................................................

*hsp70S5^8^* ........G...................................................

*hsp70S5^17^* ........G...................................................

*hsp70S5^33^* ........G...................................................

*hsp70S5^51^* ........G...................................................

*hsp70S5^63^* ........G...................................................

*hsp70S1^10^* ATTCCAAAGGTGCAAAACATGTTGCAGAACTACTTCAACGGCAAGAGCCTGAACTTGTCG

*hsp70S1^52^* .....C......................................................

*hsp70S1^71^* .....C......................................................

*hsp70S2^10^* ...............................T............................

*hsp70S2^71^* ............................................................

*hsp70S3^10^* ............................................................

*hsp70S3^33^* ............................................................

*hsp70S3^51^* ............................................................

*hsp70S3^52^* ............................................................

*hsp70S4^17^* ............................................................

*hsp70S4^52^* .....C......................................................

*hsp70S5^8^* ............................................................

*hsp70S5^17^* .....C......................................................

*hsp70S5^33^* .....C......................................................

*hsp70S5^51^* .....C......................................................

*hsp70S5^63^* ............................................................

*hsp70S1^10^* ATTAACCCGGATGAGGCGGTGGCATATGGTGCAGCAATTCAGGCCGCCATCCTGAGTGGA

*hsp70S1^52^* ............................................................

*hsp70S1^71^* ............................................................

*hsp70S2^10^* ............................................................

*hsp70S2^71^* ............................................................

*hsp70S3^10^* ............................................................

*hsp70S3^33^* ............................................................

*hsp70S3^51^* ............................................................

*hsp70S3^52^* ............................................................

*hsp70S4^17^* ............................................................

*hsp70S4^52^* ............................................................

*hsp70S5^8^* ............................................................

*hsp70S5^17^* ............................................................

*hsp70S5^33^* ............................................................

*hsp70S5^51^* ............................................................

*hsp70S5^63^* ............................................................

*hsp70S1^10^* GACAAGAGCTCAAAGATCCAAGATGTGTTGCTGGTGGATGTAGCTCCTCTGTCACTTGGT

*hsp70S1^52^* ............................................................

*hsp70S1^71^* ............................................................

*hsp70S2^10^* ............................................................

*hsp70S2^71^* ............................................................

*hsp70S3^10^* ............................................................

*hsp70S3^33^* ............................................................

*hsp70S3^51^* ............................................................

*hsp70S3^52^* ............................................................

*hsp70S4^17^* ..........................A.................................

*hsp70S4^52^* ............................................................

*hsp70S5^8^* .................T..........................................

*hsp70S5^17^* ............................................................

*hsp70S5^33^* ............................................................

*hsp70S5^51^* ..................................................T.........

*hsp70S5^63^* .................T..........................................

*hsp70S1^10^* ATCGAGACAGCAGGAGGAGTGATGACGAAGATTGTGGAGCGTAATAGTCGCATTCCGTGC

*hsp70S1^52^* ........G...................................................

*hsp70S1^71^* ........G...................................................

*hsp70S2^10^* ........G...................................................

*hsp70S2^71^* ........G...............................................T...

*hsp70S3^10^* ........G....A..............................................

*hsp70S3^33^* ........G...................................................

*hsp70S3^51^* ........G...................................................

*hsp70S3^52^* ........G...................................................

*hsp70S4^17^* ........G...................................................

*hsp70S4^52^* ........G...................................................

*hsp70S5^8^* ........G...............................................T...

*hsp70S5^17^* ........G...............................................T...

*hsp70S5^33^* ........G...................................................

*hsp70S5^51^* ........G...................................................

*hsp70S5^63^* ........G...............................................T...

*hsp70S1^10^* AAGCAGACACAAACATTCACGACATATTCAGACAACCAACCAGCAGTAACAGTTCAAGTG

*hsp70S1^52^* ..........................C.................................

*hsp70S1^71^* ..........................C.................................

*hsp70S2^10^* ..........................C.................................

*hsp70S2^71^* ..........................C.................................

*hsp70S3^10^* ..........................C.................................

*hsp70S3^33^* ..........................C.................................

*hsp70S3^51^* ..........................C.................................

*hsp70S3^52^* ..........................C.................................

*hsp70S4^17^* ............................................................

*hsp70S4^52^* ..........................C.................................

*hsp70S5^8^* ....................A.....C.................................

*hsp70S5^17^* ..........................C.................................

*hsp70S5^33^* ............................................................

*hsp70S5^51^* ............................................................

*hsp70S5^63^* ....................A.....C.................................

*hsp70S1^10^* TTCGAAGGCGAACGGGCTATGACGAAGGACAACAACTTGTTGGGCACATTCAATTTGACT

*hsp70S1^52^* ...................................T........T...............

*hsp70S1^71^* ...................................T........T...............

*hsp70S2^10^* ................................T.....A.....T...............

*hsp70S2^71^* ............................................................

*hsp70S3^10^* ...................................T........T...............

*hsp70S3^33^* ...................................T........T...............

*hsp70S3^51^* ...................................T........T...............

*hsp70S3^52^* ...................................T........T...............

*hsp70S4^17^* ............................................T...............

*hsp70S4^52^* ...................................T........T...............

*hsp70S5^8^* ............................................................

*hsp70S5^17^* ...................................T........T...............

*hsp70S5^33^* ...................................T........T...............

*hsp70S5^51^* ...................................T........T...............

*hsp70S5^63^* ............................................................

*hsp70S1^10^* GGTATTCCACCAGCACCTCGTGGAGTACCAAAGGTTGAGGTGACATTTGACTTGGACGCC

*hsp70S1^52^* A................................A..........................

*hsp70S1^71^* A................................A..........................

*hsp70S2^10^* .................................A..........................

*hsp70S2^71^* .................................A..........................

*hsp70S3^10^* .................................A..........................

*hsp70S3^33^* A................................A..........................

*hsp70S3^51^* .................................A..........................

*hsp70S3^52^* A................................A..........................

*hsp70S4^17^* ...........G.....................A..........................

*hsp70S4^52^* A................................A..........................

*hsp70S5^8^* .................................A..........................

*hsp70S5^17^* .................G...............A..........................

*hsp70S5^33^* .................................A..........................

*hsp70S5^51^* .................................A..........................

*hsp70S5^63^* .................................A..........................

*hsp70S1^10^* AATGGAATCTTGAATGTATCAGCCAAGGATACGAGCACTGGAAACTCGAAGAACATCACC

*hsp70S1^52^* ............................................................

*hsp70S1^71^* ............................................................

*hsp70S2^10^* ............................................................

*hsp70S2^71^* ............................................................

*hsp70S3^10^* ............................................................

*hsp70S3^33^* ............................................................

*hsp70S3^51^* ............................................................

*hsp70S3^52^* ............................................................

*hsp70S4^17^* ............................................................

*hsp70S4^52^* ............................................................

*hsp70S5^8^* ............................................................

*hsp70S5^17^* ............................................................

*hsp70S5^33^* ........................................................T...

*hsp70S5^51^* ........................................................T...

*hsp70S5^63^* ............................................................

*hsp70S1^10^* ATCAAGAACGACAAGGGAAGATTGTCTCAAGCTGAAATCGACAAGATGTTGGCTGAAGCT

*hsp70S1^52^* ...........................................G................

*hsp70S1^71^* ...........................................G................

*hsp70S2^10^* ............................................................

*hsp70S2^71^* ............................................................

*hsp70S3^10^* ............................................................

*hsp70S3^33^* ............................................................

*hsp70S3^51^* ............................................................

*hsp70S3^52^* ...........................................G................

*hsp70S4^17^* .................G.........................G................

*hsp70S4^52^* ...........................................G................

*hsp70S5^8^* ...........................................G................

*hsp70S5^17^* ...........................................G................

*hsp70S5^33^* ...........................................G................

*hsp70S5^51^* ...........................................G................

*hsp70S5^63^* ............................................................

*hsp70S1^10^* GAACGCTACGCGGAAGAAGACGAGAAGCAAAGGCAACGCGTGGCAGCTCGCAATCAACTT

*hsp70S1^52^* ............................................................

*hsp70S1^71^* ............................................................

*hsp70S2^10^* ............................................................

*hsp70S2^71^* ............................................................

*hsp70S3^10^* ...............................................C............

*hsp70S3^33^* ...............................................C............

*hsp70S3^51^* ...............................................C............

*hsp70S3^52^* ............................................................

*hsp70S4^17^* ...............................................C............

*hsp70S4^52^* ............................................................

*hsp70S5^8^* ...............................................C............

*hsp70S5^17^* ...............................................C............

*hsp70S5^33^* ...............................................C............

*hsp70S5^51^* ............................................................

*hsp70S5^63^* ...............................................C............

*hsp70S1^10^* GAAGGATATGTGTTCAATGTGAAACAGTCAGTGGAAGATGCTGGTGACAAGTTGTCTCAG

*hsp70S1^52^* .................................C..........A...............

*hsp70S1^71^* .................................C..........A...............

*hsp70S2^10^* .......................G....................................

*hsp70S2^71^* ............................................................

*hsp70S3^10^* ............................................A...............

*hsp70S3^33^* ............................................A...............

*hsp70S3^51^* ............................................A...............

*hsp70S3^52^* .................................C..........A...............

*hsp70S4^17^* ............................................................

*hsp70S4^52^* ............................................................

*hsp70S5^8^* ............................................A...............

*hsp70S5^17^* ............................................A...............

*hsp70S5^33^* ..............T...........A.................A...............

*hsp70S5^51^* ..............T...........A.................A...............

*hsp70S5^63^* ............................................A...............

*hsp70S1^10^* TCAGATAAGAACACAGTGCAGAAAGCTTGCGAGGACACAATTAAGTGGTTGGATAACAAT

*hsp70S1^52^* ...................T....................................T...

*hsp70S1^71^* ...................T..................T.................T...

*hsp70S2^10^* ............................................................

*hsp70S2^71^* ............................................................

*hsp70S3^10^* ..C.........................................................

*hsp70S3^33^* ..C......................T..................................

*hsp70S3^51^* ..C.........................................................

*hsp70S3^52^* ...................T....................................T...

*hsp70S4^17^* ............................................................

*hsp70S4^52^* .................A.T..................T.................T...

*hsp70S5^8^* ..C..............A.T........................................

*hsp70S5^17^* ...................TA..................................G....

*hsp70S5^33^* ..C................TA.......................................

*hsp70S5^51^* ..C................TA.......................................

*hsp70S5^63^* ..C..............A.T........................................

*hsp70S1^10^* AACTTGGCTGACAAGGAGGAGTTTGAACACCGAATGCAAGAACTTACCCGACAATGCAGT

*hsp70S1^52^* ..............A.............................................

*hsp70S1^71^* ..............A.............................................

*hsp70S2^10^* ............................................................

*hsp70S2^71^* ............................................................

*hsp70S3^10^* ............................................................

*hsp70S3^33^* ............................................................

*hsp70S3^51^* ............................................................

*hsp70S3^52^* ..............A.............................................

*hsp70S4^17^* ..............A.............................A...............

*hsp70S4^52^* .........A....A...................................G.........

*hsp70S5^8^* ............................C...............................

*hsp70S5^17^* ..............A.....A.......................................

*hsp70S5^33^* ............................................A...............

*hsp70S5^51^* ............................................A...............

*hsp70S5^63^* ............................................................

*hsp70S1^10^* CCGATAATGACGAAGTTGCACACAGGCGGTGCTCAGCCACAAGGAGGTAGTTGCGGCCAA

*hsp70S1^52^* ............................................................

*hsp70S1^71^* ............................................................

*hsp70S2^10^* ....................................T.......................

*hsp70S2^71^* ....................................T.......................

*hsp70S3^10^* .............................................A..............

*hsp70S3^33^* .............................................A..............

*hsp70S3^51^* .............................................A..............

*hsp70S3^52^* .............................................A..............

*hsp70S4^17^* ....................................T.......................

*hsp70S4^52^* ....................................T.......................

*hsp70S5^8^* ............................................................

*hsp70S5^17^* ............................................................

*hsp70S5^33^* ............................................................

*hsp70S5^51^* ..........................................C.................

*hsp70S5^63^* ............................................................

*hsp70S1^10^* CAGGCAGGTGGGTTTGGAGGAGGACGCTCAGGTCCGACAGTTGAGGAAGTAGATTAA

*hsp70S1^52^* .........................................................

*hsp70S1^71^* .........................................................

*hsp70S2^10^* ...........A..C..............T...........................

*hsp70S2^71^* ...........A..C..............T...........................

*hsp70S3^10^* ...........A..C..............T...........................

*hsp70S3^33^* ...........A..C..............T...........................

*hsp70S3^51^* ...........A..C..............T...........................

*hsp70S3^52^* ...........A..C..............T...........................

*hsp70S4^17^* ...........A..C..............T...........G...........C...

*hsp70S4^52^* ...........A..C..............T...........G...........C...

*hsp70S5^8^* .....T....................A..T...........G...............

*hsp70S5^17^* .....T.....C..............A..T...........................

*hsp70S5^33^* .....T....................A..T...........................

*hsp70S5^51^* .....T....................A..T...........................

*hsp70S5^63^* .....T....................A..T...........................
