# Supplementary material for: Organization and evolution of hsp70 clusters strikingly differ in two species of Stratiomyidae (Diptera) inhabiting thermally contrasting environments
Source: BMC Evol Biol. 2011 Mar 22;11:74. doi: 10.1186/1471-2148-11-74 (PMC3071340; doi:10.1186/1471-2148-11-74)
Supplement: Additional file 9 — Figure S7. Alignment of Stratiomys hsp70 5'-UTR sequences. [file 1471-2148-11-74-S9.DOC]

**Additional file 9: Figure S7. Alignment of *Stratiomys hsp70* 5’-UTR sequences.** Sequences begin at TATA box and end on last nucleotide before start codon. Alleles named by phage number (superscript). Dots indicated identical nucleotides. Black bars indicate conversion-mediated shared SNPs. Grey bars mark uninterrupted conversion tracts. Triangle marks insertion of unaligned repetitive sequence in *hsp70S3^71^*.

*hsp70S1^52^* TATATATACTACCAGTCGGCCAACATGAGCTCAGTAATAAACTGTAAAACAAACGAAACA

*hsp70S1^10^* .............................T............................T.

*hsp70S1^71^* ............................................................

*hsp70S2^10^* .............................T..............................

*hsp70S2^71^* .............................T..............................

*hsp70S3^10^* .............................T..............................

*hsp70S3^33^* .............................T..............................

*hsp70S3^51^* .............................T..............................

*hsp70S3^52^* .............................T..............................

*hsp70S3^5^* .............................T..............................

*hsp70S4^52^* .............................T..............................

*hsp70S4^5^* .............................T..............................

*hsp70S4^17^* .............................T..............................

*hsp70S5^17^* .............................T..............................

*hsp70S5^33^* .............................T..............................

*hsp70S5^51^* .............................T..............................

*hsp70S5^63^* ....................................................G.A..G..

*hsp70S5^8^* ....................................................G.A..G..

*hsp70S1^52^* CTTTAATAGAGTGAAACAAGCGAGAACGAGTGAATCTTATTCGAATTTCAAATAGTGAAC

*hsp70S1^10^* ...C..C.....................................................

*hsp70S1^71^* ............................................................

*hsp70S2^10^* ...C........................................................

*hsp70S2^71^* ...C..C.....................................................

*hsp70S3^10^* ...C........................................................

*hsp70S3^33^* ...C........................................................

*hsp70S3^51^* ...C........................................................

*hsp70S3^52^* ...C..C.....................................................

*hsp70S3^5^* ...C..C.............................................A.......

*hsp70S4^52^* ...C..C.........A.....................TA....................

*hsp70S4^5^* ...C..C.............................................A.......

*hsp70S4^17^* ...C........................................................

*hsp70S5^17^* T..C................................................A.......

*hsp70S5^33^* T..C...................A........................A...A.......

*hsp70S5^51^* T..C...................A........................A...A.......

*hsp70S5^63^* ...G................................................A.......

*hsp70S5^8^* ...G................................................A.......

*hsp70S1^52^* AACCGAAAAATTGTGAAAGGATTATTGAGTTATCAATCGAAGAATAAGTGAAAGTTGAAA

*hsp70S1^10^* ...................C....CC...........T......................

*hsp70S1^71^* ............................................................

*hsp70S2^10^* ...................C....CC...........T......................

*hsp70S2^71^* .........G.........C..A.CC..................................

*hsp70S3^10^* ........C..........C....CC...........T......................

*hsp70S3^33^* ........C..........C....CC...........T......................

*hsp70S3^51^* ........C..........C....CC...........T......................

*hsp70S3^52^* .........G.........C..A.CC..................................

*hsp70S3^5^* ........................A.................G.................

*hsp70S4^52^* ..A..G......................................................

*hsp70S4^5^* ........................A.................G.................

*hsp70S4^17^* ...................C....CC...........T......................

*hsp70S5^17^* .....G......................................................

*hsp70S5^33^* ............................................................

*hsp70S5^51^* ............................................................

*hsp70S5^63^* .....G..................CC..................................

*hsp70S5^8^* .....G..................CC..................................

*hsp70S1^52^* AGAAGTTAAAATTTTAATTCATCAATACAAAGGACTATTAAGTTATCAGTTGAAGAATTA

*hsp70S1^10^* ......G..................C..................................

*hsp70S1^71^* ............................................................

*hsp70S2^10^* ......G........................A............................

*hsp70S2^71^* ..........................................................A.

*hsp70S3^10^* ......G..................C..................................

*hsp70S3^33^* ......G..................C..................................

*hsp70S3^51^* ......G..................C..................................

*hsp70S3^52^* ..........................................................A.

*hsp70S3^5^* ......G.....................................G...............

*hsp70S4^52^* ............................................................

*hsp70S4^5^* ......G.....................................G...............

*hsp70S4^17^* ......G...G...G..A..G................A......................

*hsp70S5^17^* ......G..................C..................................

*hsp70S5^33^* ............................................................

*hsp70S5^51^* ............................................................

*hsp70S5^63^* ......G...G...G..A..G................A......................

*hsp70S5^8^* ......G...G...G..A..G................A......................

*hsp70S1^52^* ACTGCAAAAGAAAAGTTGAAAA

*hsp70S1^10^* ......................

*hsp70S1^71^* ......................

*hsp70S2^10^* ...CTC................

*hsp70S2^71^* GTGAACG..ATC.GT..A....

*hsp70S3^10^* ....A.................

*hsp70S3^33^* ...CTC................

*hsp70S3^51^* ....A.................

*hsp70S3^52^* GTGAACG..ATC.GT..A....

*hsp70S3^5^* ...CTC................

*hsp70S4^52^* ......................

*hsp70S4^5^* ...CTC................

*hsp70S4^17^* ...CTC................

*hsp70S5^17^* ....A.................

*hsp70S5^33^* ...CTC................

*hsp70S5^51^* ...CTC................

*hsp70S5^63^* ...CTC................

*hsp70S5^8^* ...CTC................

TE remnants

*hsp70S3^71^*

*hsp70S5^71^* ...............
