# Supplementary material for: Organization and evolution of hsp70 clusters strikingly differ in two species of Stratiomyidae (Diptera) inhabiting thermally contrasting environments
Source: BMC Evol Biol. 2011 Mar 22;11:74. doi: 10.1186/1471-2148-11-74 (PMC3071340; doi:10.1186/1471-2148-11-74)
Supplement: Additional file 10 — Figure S8. Alignment of hsp70S1 3'-UTR sequences. [file 1471-2148-11-74-S10.DOC]

**Additional file 10: Figure S8. Alignment of *hsp70S1* 3’-UTR sequences.** Sequences begin on first nucleotide after stop codon. Alleles named by phage number (superscript). Dots indicated identical nucleotides, dashes are gaps.

*hsp70S1^10^* GCAAATGTCGACAGATTCCTAATAGTGATAAGTTCCGAATGGATAATTTGA--AGTTGCG

*hsp70S1^71^* ......A...--.....................................AGGT.......

*hsp70S1^52^* ......A...-----------------------------------------..-------

*hsp70S1^10^* GCCAACAGGCAGGTGGGTTTGGAGGAGGACGCTCAGGTCCGACAGTTGAGGAACGACAAT

*hsp70S1^71^* .................................................TACC.......

*hsp70S1^52^* ------------------------------------------------------------

*hsp70S1^10^* GCAGTCCGATAATGACGAAGTTGCACACAGGCGGTGCTCAGCCACAAGG

*hsp70S1^71^* .................................................

*hsp70S1^52^* ------------.....................................
