# Supplementary material for: Organization and evolution of hsp70 clusters strikingly differ in two species of Stratiomyidae (Diptera) inhabiting thermally contrasting environments
Source: BMC Evol Biol. 2011 Mar 22;11:74. doi: 10.1186/1471-2148-11-74 (PMC3071340; doi:10.1186/1471-2148-11-74)
Supplement: Additional file 11 — Figure S9. Alignment of hsp70S2 and hsp70S3 3'-UTR sequences. [file 1471-2148-11-74-S11.DOC]

**Additional file 11: Figure S9. Alignment of *hsp70S2* and *hsp70S3* 3’-UTR sequences.** Sequences begin on first nucleotide after stop codon. Alleles named by phage number (superscript). Dots indicated identical nucleotides, dashes are gaps. Grey bars mark uninterrupted conversion tracts.

*hsp70S2^71^* GCAAATGTCGACAGATTCCTAATAGTGATAAGTTCCGAATGGATAATTTGAATGAGATGT

*hsp70S2^10^* ............................................................

*hsp70S2^5^* ...........--.....................TT....T...........C.......

*hsp70S3^10^* ............................................................

*hsp70S3^5^* ...........--.....................TT....T...........C.......

*hsp70S3^17^* ...........--.....................TT....T...........C.......

*hsp70S3^33^* ...........--.....................TT....T...........C.......

*hsp70S3^51^* ...........--.....................TT....T...........C.......

*hsp70S3^52^* ...........--....................................A..C.......

*hsp70S2^71^* TTGAATTAAGAGTATTGAATAATTTGTTATATATTGATTAGCTTGAAGACTGTTAGTATT

*hsp70S2^10^* ............................................................

*hsp70S2^5^* ......GT......A.............G...........CT..................

*hsp70S3^10^* ............................................................

*hsp70S3^5^* ......GT......A.............G...........CT..................

*hsp70S3^17^* ......GT......A.............G...........CT..................

*hsp70S3^33^* ......GT......A.............G...........CT..................

*hsp70S3^51^* ......GT......A.............G...........CT..................

*hsp70S3^52^* ......GT......A.........................CT..................

*hsp70S2^71^* AATGATTTATTGAATATTTTATAT------TATTGTAATTGA----GTAGTATGTGACCT

*hsp70S2^10^* ............................................................

*hsp70S2^5^* .T.A..............G..AG.ACAATG.GA.A...A...TATG...T...AAA.TG.

*hsp70S3^10^* ............................................................

*hsp70S3^5^* .T.A..............G..AG.ACAATG.GA.A...A...TATG...T...AAA.TG.

*hsp70S3^17^* .T.A..............G..AG.ACAATG.GA.A...A...TATG...T...AAA.TG.

*hsp70S3^33^* .T.A..............G..AG.ACAATG.GA.A...A...TATG...T...AAA.TG.

*hsp70S3^51^* .T.A..............G..AG.ACCATG.GA.A...A...TATG...T...AAA.TG.

*hsp70S3^52^* .T.A..............G..AG.ACGATG.GA.A...A...TATG...T...AAA.TG.

*hsp70S2^71^* ATTCATGTAATG------------------TGTCTTCTTTTGAAGGCGTTAATAAAA-TA

*hsp70S2^10^* ............................................................

*hsp70S2^5^* .AA....ATG..CAAAAACATGTCATGTCA..........C.......A........A..

*hsp70S3^10^* ............................................................

*hsp70S3^5^* .AA....ATG..CAAAAACATGTCATGTCA..........C.......A........A..

*hsp70S3^17^* .AA....ATG..CAAAAACATGTCATGTCA..........C.......A........A..

*hsp70S3^33^* .AA....ATG..CAAAAACATGTCA...............C.......A........A..

*hsp70S3^51^* .AA....ATG..CAAAAACATGTCATGTCA..........C.......A........A..

*hsp70S3^52^* .AA....ATG..CAAAAACATGTCA...............C.......A........A..

*hsp70S2^71^* ATTTAAATGAAA--TTAATT---GTTTTCTCTCTTAGCT---GGTACGGTTCTGAATTAG

*hsp70S2^10^* ............................................................

*hsp70S2^5^* ....G..CT...AA.C.C..TGG....AT.A.T...TG.TTT.T.T.TCC.T.TT...CA

*hsp70S3^10^* ............................................................

*hsp70S3^5^* ....G..CT...AA.C.C..TGG....AT.A.T...TG.TTT.T.T.TCC.T.TT...CA

*hsp70S3^17^* ....G..CT...A..C.C...GG....AT.A.T...TG.TT..T.T.TCC.T.TT...CA

*hsp70S3^33^* ....G..CT...A..C.C...GG....AT.A.T...TG.TT..T.T.TCC.T.TT...CA

*hsp70S3^51^* ....G..CT...A..C.C...GG....AT.A.T...TG.TT..T.T.TCC.T.TT...CA

*hsp70S3^52^* ....G..CT...A..C.C...GG....AT.A.T...TG.TT..T.T.TCC.T.TT...CA

*hsp70S2^71^* GGTTCGCCAGTTTTATTTTAAGAGCAA--CGCGGCTACAATTGAATATTCAA-TTCGT-T

*hsp70S2^10^* ............................................................

*hsp70S2^5^* A...GAT.GTC....C...T....T..AT.A.CA...TT.G...T.GA.A..A..AACC.

*hsp70S3^10^* ............................................................

*hsp70S3^5^* A...GAT.GTC....C...T....T..AT.A.-A...T-.G...T.GA.A.....AAC..

*hsp70S3^17^* A...GAT.GTC....C...T....T..AT.A.-A...T-.G...T.GA.A.....AAC..

*hsp70S3^33^* A...GAT.GTC...GC...T....T..AT.A.-A...T-.G...T.GA.A.....AAC..

*hsp70S3^51^* A...GAT.GTC....C...T....T..AT.A.-A...T-.G...T.GA.A.....AAC..

*hsp70S3^52^* A...GAT.GTC...GC...T....T..AT.A.-A...T-.G...T.GA.A.....AAC..

*hsp70S2^71^* GATCT

*hsp70S2^10^* .....

*hsp70S2^5^* A.C..

*hsp70S3^10^* .....

*hsp70S3^5^* AC...

*hsp70S3^17^* AC...

*hsp70S3^33^* AC...

*hsp70S3^51^* AC...

*hsp70S3^52^* AC...
