# Supplementary material for: Organization and evolution of hsp70 clusters strikingly differ in two species of Stratiomyidae (Diptera) inhabiting thermally contrasting environments
Source: BMC Evol Biol. 2011 Mar 22;11:74. doi: 10.1186/1471-2148-11-74 (PMC3071340; doi:10.1186/1471-2148-11-74)
Supplement: Additional file 12 — Figure S10. Alignment of hsp70S4 3'-UTR sequences. [file 1471-2148-11-74-S12.DOC]

**Additional file 12: Figure S10. Alignment of *hsp70S4* 3’-UTR sequences.** Sequences begin on first nucleotide after stop codon. Alleles named by phage number (superscript). Dots indicated identical nucleotides, dashes are gaps.

*hsp70S4^52^* TTCCCTCATAATGTTAAGTAGAATTTAGATTGTGTGAATGAGTGAATATTCGAAGGATGT

*hsp70S4^17^* ............A...............................................

*hsp70S4^52^* ATGAGTTTATACCGCAGCATAGGTCGGAATTTGTAATTTATTGAAAATTTAGTTGTAACA

*hsp70S4^17^* T........A....A...........A.........................C.......

*hsp70S4^52^* TTAAATTTCTTAATGTACATTAAGATGAAAATCGTGACTGTTTTGTCTTCTTGTGAAGAC

*hsp70S4^17^* .........................A..................................

*hsp70S4^52^* GGAAATAAACTACTTTAAACACAAGTAATTTGGACTTGTTATTTAGCATTATTGTTTTCA

*hsp70S4^17^* .....................................A............G.........

*hsp70S4^52^* GACTGTTTAATCTAATGCTCATGACATATTCCTGTTCAGCTGGTATAACTTCGTTTGTAT

*hsp70S4^17^* ............................................................

*hsp70S4^52^* ATTTTTTTGGTAAAAAAAGGG-GGAAGCAGGCGCTTCATTTTGTTTCAGTTTTCTTTTCT

*hsp70S4^17^* ..................A..C..................A...................

*hsp70S4^52^* GGGTCAGTTCACATGTAATAATGCTAACGTTGTTACGTTTTTTAGTGTTTGTTGTACCTA

*hsp70S4^17^* ............................................................

*hsp70S4^52^* TAAGCAAGTCAGGCAGTTTGATTGGGTTATCAAAGGTAGTTTTACTATTGTTGTCAATTT

*hsp70S4^17^* ....---------.......................C...G...................

*hsp70S4^52^* CCTCGGATAATTTGAAGGCTCTGTATAGCATTTGCCAGCCCATTGTCTACGATCTCAGTT

*hsp70S4^17^* .-....G.....................................................

*hsp70S4^52^* CATTTATTATTTCTATGTTTCTAACTATCGTTA-----------------------ATTT

*hsp70S4^17^* .....................C.CG.T.TAA.GTTCTATGTTTCTATGTTTTCCATG...

*hsp70S4^52^* TTAGATTGCAGAAGCAGATTCCTCTTGTCCATTCTGGTTTT

*hsp70S4^17^* .A................A......................
