# Supplementary material for: Organization and evolution of hsp70 clusters strikingly differ in two species of Stratiomyidae (Diptera) inhabiting thermally contrasting environments
Source: BMC Evol Biol. 2011 Mar 22;11:74. doi: 10.1186/1471-2148-11-74 (PMC3071340; doi:10.1186/1471-2148-11-74)
Supplement: Additional file 13 — Figure S11. Alignment of hsp70S5 3'-UTR sequences. [file 1471-2148-11-74-S13.DOC]

**Additional file 13: Figure S11. Alignment of *hsp70S5* 3’-UTR sequences.** Sequences begin on first nucleotide after stop codon. Alleles named by phage number (superscript). Dots indicated identical nucleotides, dashes are gaps.

*hsp70S5^17^* GCAAATTTTGAGACCCCTAATAGTGATGTTCTGAATGGATAATTTGAATGAGATGTTTGA

*hsp70S5^33^* ............................................................

*hsp70S5^51^* ............................................................

*hsp70S5^63^* ............................................................

*hsp70S5^8^* ............................................................

*hsp70S5^17^* ATGTAAAATATTGAATAATTTGTTTTATATTGATTAGTTTGAAGACTGTTAGTATTAATA

*hsp70S5^33^* ............................................................

*hsp70S5^51^* ............................................................

*hsp70S5^63^* ...........................C........T.......................

*hsp70S5^8^* ...........................C........T.......................

*hsp70S5^17^* ATTTATTGAATATTGTAAGTCTGATGCATCTTAATAAGTTAAAATCAAAGGTGAAAATAA

*hsp70S5^33^* ............................................................

*hsp70S5^51^* ............................................................

*hsp70S5^63^* ............................................................

*hsp70S5^8^* ............................................................

*hsp70S5^17^* ATTTATTTAAATGAAACTTCCATCTGTTACTTCTGATTTGATTCCATTTGATCTGCTTCC

*hsp70S5^33^* .....................G......................................

*hsp70S5^51^* .....................G......................................

*hsp70S5^63^* .....................G......................................

*hsp70S5^8^* .....................G......................................

*hsp70S5^17^* AATGTAGCAGTCAGACGTTCTTTAAGTGCACGCAACGTACACTCAGTTTGTAGGTATCAA

*hsp70S5^33^* ............................................................

*hsp70S5^51^* ............................................................

*hsp70S5^63^* ..............................T.............................

*hsp70S5^8^* ............................................................

*hsp70S5^17^* GAATTAAATGGTGAATAGAGTAGACAAAAGTATACACTATACAATGTGATATATAAAATT

*hsp70S5^33^* ............................................................

*hsp70S5^51^* ....G.......................................................

*hsp70S5^63^* ..................................G.........................

*hsp70S5^8^* ..................................G.........................

*hsp70S5^17^* TATTGTTGAATGTTTTTAGTTATGAACGTATCATTGCAGTGTTGCTTTCCTAAG-TGGAT

*hsp70S5^33^* ...............................................G......G.....

*hsp70S5^51^* ...............................................G......G.....

*hsp70S5^63^* ......................................................G.....

*hsp70S5^8^* ................A.....................................G.....

*hsp70S5^17^* GAAATGGTTAAATTCGCTAAATGTGTTTTA-TTATCAGTAATCGGAAATCGATTAAATAC

*hsp70S5^33^* .................G......................................TACG

*hsp70S5^51^* .................G............A.........................TACG

*hsp70S5^63^* .................G......................................TACG

*hsp70S5^8^* .................G......................................TACG

*hsp70S5^17^* AGTGTTTGATCTTCCAAGACCTGCTTGGGAATACAACCTTACTCTTTTGAAATCTAGA

*hsp70S5^33^* ................................................TG........

*hsp70S5^51^* ................................................-G........

*hsp70S5^63^* ................................................TG........

*hsp70S5^8^* ................................................TG........
