# Supplementary material for: Organization and evolution of hsp70 clusters strikingly differ in two species of Stratiomyidae (Diptera) inhabiting thermally contrasting environments
Source: BMC Evol Biol. 2011 Mar 22;11:74. doi: 10.1186/1471-2148-11-74 (PMC3071340; doi:10.1186/1471-2148-11-74)
Supplement: Additional file 14 — Figure S12. Deduced amino acids sequence of Hsp70S1 (S. singularior), Hsp70P1 and Hsp68 (both from O. pardalina). Comparison with paralogs from several organisms exhibiting homology to Stratiomyidae Hsp70. [file 1471-2148-11-74-S14.DOC]

**Additional file 14: Figure S12. Deduced amino acids sequence of Hsp70S1 (*S. singularior*), Hsp70P1 and Hsp68 (both from *O. pardalina*).** Different and sometimes overlapping peptides determined by fingerprinting analysis from the correspondent Hsp70 family proteins excised from 2D gels are underlined. Paralogs from several organisms exhibiting homology to Stratiomyidae Hsp70 (Figure 6) are taken for comparison.

1 ▼ ▼ 70

Hsp70S1 S.sin (1) **M----PAVGIDLGTTFSCVGVFQHGK**VEIIANDQGNR**TTPSYVAFTDSER**LIGDAAK**NQVAMNPKNTVFD**

Hsp70P1 O.par (1) **M----PAVGIDLGTTFSCVGVFQHGKVEIIANDQGN**R**TTPSYVAFTESER**LIGDAAK**NQVAMNPKNTVFD**

Hsp68 O.par (1) M----PAIGIDLGTTYSCVGVFQHGKVEIIANDQGNRTTPSYVAFTDSERLIGDAAKNQVAMNPKNSVFD

Hsp70 B.ole (1) M----VAIGIDLGTTYSCVGVFQHGKVEIIANDQGNRTTPSYVAFTDSERLIGDAAKNQVAMNPKNTVFD

Hsp70Ab A.aeg (1) M----SAIGIDLGTTYSCVGVFQHGKVEIIANDQGNRTTPSYVSFSDTERLIGDAAKNQVALNPQNTVFD

Hsp70 B.mor (1) M----PAIGIDLGTTYSCVGVWQHGNVEIIANDQGNRTTPSYVAFTDTERLIGDAAKNQVALNPNNTVFD

Hsp70 M.bra (1) M----PAIGIDLGTTYSCVGVWQHGNVEIIANDQGNRTTPSYVAFTDTERLIGDAAKNQVALNPSNTVFD

Hsp70 R.pom (1) M----VAIGIDLGTTYSCVGVFQHGKVEIIANDQGNRTTPSYVAFTDSERLIGDAAKNQVAMNPKNTVFD

Hsp70 L.mig (1) MAVKAPAVGIDLGTTYSCVGVFQHGKVEIIANDQGNRTTPSYVAFTDTERLIGDAAKNQVAMNPSNTIFD

Hsp70 L.sat (1) M----PAIGIDLGTTYSCVGVFQHGKVEIIANDQGNRTTPSYVAFTDSERLIGDAAKNQVAMNPKNSVFD

Hsp70 D.moj (1) M----PAIGIDLGTTYSCVGVFQHGKVEIIANDQGNRTTPSYVAFTDSERLIGDAAKNQVAMNPSNTVFD

Hsp70 D.vir (1) M----PAIGIDLGTTYTCVGVYQHGKVEIIANDQGNRTTPSYVAFTDSERLIGDAAKNQVAMNPKNTVFD

Hsp70 D.ana (1) M----PAIGIDLGTTYSCVGVYQHGKVEIIANDQGNRTTPSYVAFTESERLIGDAAKNQVAMNPRNTVFD

Hsp70Aa D.mel (1) M----PAIGIDLGTTYSCVGVYQHGKVEIIANDQGNRTTPSYVAFTDSERLIGDPAKNQVAMNPRNTVFD

Hsp70 D.ant (1) M----VAIGIDLGTTYSCVGVFQHGKVEIIANDQGNRTTPSYVAFTDSERLIGDAAKNQVAMNPQNTVFD

* * ******* **** *** ***************** * ****** ****** ** * **

71 ▼ ▼ ▼ 140

Hsp70S1 S.sin (67) **AKR**LIGRKYDDPKIQEDLRHWPFTVKSDSGKPKICVEFKGEQK**RFAPEEISSMVLTKMK**ETAEAYLGESV

Hsp70P1 O.par (67) **AKR**LIGRKYDDPKIQEDLRNWPFTVKSDSGKPKICVEFKGEQK**RFAPEEISSMVLTKMKETAEAYLGETV**

Hsp68 O.par (67) AKRLIGRKFEDPKIQDDIKHWPFKVVSDGGKPKIVVEFKGEIKKFAPEEISSMVLTKMKDTAEAYLGSTI

Hsp70 B.ole (67) AKRLIGRKYDDPKIMEDVKHWPFKVVSDGGKPKISVEYKGENKRFAPEEISSMVLTKMKETAEAYLGTTV

Hsp70Ab A.aeg (67) AKRLIGRRFDDPKIQADIKHWPFKVFNDAGKPKIEVEFKGEKKRFAPEEISSMVLVKMRETAEAYLGKSV

Hsp70 B.mor (67) AKGLIGRKFDDPKIQQDMKHWPFKVINDCGKPKIQIEFKGETKRFAPEEISSMVLTKMKETAEAYLGSTV

Hsp70 M.bra (67) AKRLIGRKFDDSKIQQDMKHWPFKVVNECGKPKIVVDFKGEAKRFAPEEISSMVLTKMKETAEAYLGSSV

Hsp70 R.pom (67) AKRLIGRKYDDPKIMEDIKHWPFKVVSDGGKPKICVEYKGENKRFAPEEISSMVLTKMKETAEAYLGTTV

Hsp70 L.mig (71) AKRLIGRRFDDQAVQSDMKHWPFKVINDSGKPKIQVQYKGETKTFFPEEVSSMVLTKMKETAEAYLGKNV

Hsp70 L.sat (67) AKRLIGRKFDDPKIQSDMKHWPFKVVNDCGKPKICVEFKGEEKKFAPEEISSMVLTKMKETAEAFLGTTI

Hsp70 D.moj (67) AKRLIGRKYDDPKIQSDMKHWPFKVVNDCGKPKINVEFKGEQKRFAPEEISSMVLVKMKETAEAYLGTTI

Hsp70 D.vir (67) AKRLIGRRYDDPKIAEDIKHWPFKVVSDGGKPKIGVQFKGEQKRFAPEEISSMVLVKMKETAEAYLGQSI

Hsp70 D.ana (67) AKRLIGRKYDDPKIAEDIKHWPFKVVSDGGKPKIGVEFKGEPKRFAPEEISSMVLVKMKETAEAYLGETV

Hsp70Aa D.mel (67) AKRLIGRKYDDPKIAEDMKHWPFKVVSDGGKPKIGVEYKGESKRFAPEEISSMVLTKMKETAEAYLGESI

Hsp70 D.ant (67) AKRLIGRKFDDQKIQEDMKHWPFKVINDCGKPKISVEFKGEQRKFAPEEISSMVLTKMKETAEAFLGTTV

** **** * * *** * ***** *** * *** ***** ** **** **

141 210

Hsp70S1 S.sin (137) TDAVITVPAYFNDSQRQATK**DAGAIAGLNVLRIINEPTAAALAYGLDK**NLKGERNVLIFDLGGGTFDVSI

Hsp70P1 O.par (137) **SDAVITVPAYFNDSQR**QATK**DAGAIAGLNVLRIINEPTAAALAYGLDK**NLSGERNVLIFDLGGGTFDISI

Hsp68 O.par (137) KDAVITVPAYFNDSQRQATKDAGAIAGINVLRIINEPTAAALAYGLDKNLSGERNVLIFDLGGGTFDVSI

Hsp70 B.ole (137) TDAVITVPAYFNDSQRQATKDAGRIAGLNVLRIINEPTAAALAYGLDKNLKGERNVLIFDLGGGTFDVSI

Hsp70Ab A.aeg (137) KNAVITVPAYFNDSQRQATKDAGAIAGLNVMRIINEPTAAALAYGLDKNLKGERNVLIFDLGGGTFDVSI

Hsp70 B.mor (137) RDAVVTVPAYFNDSQRQATKDAGAIAGLNVLRIINEPTAAALAYGLDKNLKGERNVLIFDLGGGTFDVSI

Hsp70 M.bra (137) RDAVITVPAYFNDSQRQATKDAGAIAGLNVLRIINEPTAAALAYGLDKNLKGERNVLIFDLGGGTFDVSI

Hsp70 R.pom (137) TDAVITVPAYFNDSQRQATKDAGRIAGLNVLRIINEPTAAALAYGLDKNLKGERNVLIFDLGGGTFDVSI

Hsp70 L.mig (141) SNAVITVPAYFNDSQRQATKDAGAIAGLNVLRIINEPTAAAIAYGLDKKGHGERNVLIFDLGGGTFDVSI

Hsp70 L.sat (137) KDAVITVPAYFNDSQRQATKDAGAIAGLNVLRIINEPTAAALAYGLDKNLKGEKNVLIFDLGGGTFDVSI

Hsp70 D.moj (137) RDAVITVPAYFNDSQRQATKDAGAIAGLNVLRIINEPTAAALAYGLDKNLKGERNVLIFDLGGGTFDVSI

Hsp70 D.vir (137) TDAVITVPAYFNDSQRQATKDAGHIAGLNVLRIINEPTAAALAYGLDKNLKGERNVLIFDLGGGTFDVSI

Hsp70 D.ana (137) SDAVITVPAYFNDSQRQATKDAGRIAGLNVLRIINEPTAAALAYGLDKNLKGERNVLIFDLGGGTFDVSI

Hsp70Aa D.mel (137) TDAVITVPAYFNDSQRQATKDAGHIAGLNVLRIINEPTAAALAYGLDKNLKGERNVLIFDLGGGTFDVSI

Hsp70 D.ant (137) RDAVVTVPAYFNDSQRQATKDAGAIAGLNVLRIINEPTAAALAYGLDKNLKGERNVLIFDLGGGTFDVSI

** ****************** *** ** ********** ****** ** ************* **

211 ▼ 280

Hsp70S1 S.sin (207) LTIDEGSLFEVR**ATAGDTHLGGEDFDNR**LVSYLADEFKRKYKKDLRSNPRALRRLRTAAERAKRTLSSST

Hsp70P1 O.par (207) LTIDEGSLFEVR**ATAGDTHLGGEDFDNR**LVSHLADEFKRKFKKDLRSNPRALRRLRTAAERAKRTLSSST

Hsp68 O.par (207) LTIDEGSLFEVKSTAGDTHLGGEDFDNRLVDHLAEEFKRKFKKDLKTNPRALRRLRTAAERAKRTLSSST

Hsp70 B.ole (207) LTIDEGSLFEVRATAGDTHLGGEDFDNRLVNHLADEFKRKYKKDLRSNPRALRRLRTAAERAKRTLSSST

Hsp70Ab A.aeg (207) LTIDEGSLFEVRATAGDTHLGGEDFDNRMVSHFVEEFKRKFKKDISNNPRALRRLRIACERAKRTLSSST

Hsp70 B.mor (207) LTIDEGSLFEVKSTAGDTHLGGEDFDNRLVNHLAEEFKRKYKKDLRLNSRALRRLRTAAERAKRTLSSST

Hsp70 M.bra (207) LTIDEGSLFEVRATAGDTHLGGEDFDNRLVNHLADEFKRKYKKDMSMNPRALRRLRTAAERAKRTLSSST

Hsp70 R.pom (207) LTIDEGSLFEVRATAGDTHLGGEDFDNRLVNHFAEEFKRKYKKDLRTNPRALRRLRTAAERAKRTLSSST

Hsp70 L.mig (211) LTIEDG-IFEVKATAGDTHLGGEDFDNRMVNHFVQEFKRKYKKDLTTNKRALRRLRTACERAKRTLSSST

Hsp70 L.sat (207) LTIDEGSLFEVRSTAGDTHLGGEDFDNRLVNHLADEFKRKYKKDLRSNPRALRRLRTAAERAKRTLSSST

Hsp70 D.moj (207) LTIDEGSLFEVRATAGDTHLGGEDFDNRLVNHLADEFKRKFKKDLRSNPRALRRLRTAAERAKRTLSSST

Hsp70 D.vir (207) LTIDEGSLFEVRSTAGDTHLGGEDFDNRLVTHLAEEFKRKYKKDLRSNPRALRRLRTAAERAKRTLSSST

Hsp70 D.ana (207) LTIDEGSLFEVRATAGDTHLGGEDFDNRLVTHLAEEFKRKFKKDLRSNPRALRRLRTAAERAKRTLSSST

Hsp70Aa D.mel (207) LTIDEGSLFEVRSTAGDTHLGGEDFDNRLVTHLADEFKRKYKKDLRSNPRALRRLRTAAERAKRTLSSST

Hsp70 D.ant (207) LTIDEGSLFEVRATAGDTHLGGEDFDNRLVTHLAEEFKRKYKKDLRSNPRALRRLRSAAERAKRTLSSST

*** * *** *************** * ***** *** * ******* * ***********

281 350

Hsp70S1 S.sin (277) ETTIEIDALYEGVDFYTKVSRAR**FEELCADLFR**STLQPVEKALNDAKMDKSQIHDIVMVGGSTRIPKVQN

Hsp70P1 O.par (277) EATIEIDALFEGADFYTKVS**RARFEELCADLFR**STLQPVEKALCDAKMDKAQIHDIVLVGGSTRIPKVQS

Hsp68 O.par (277) EASIEIDALLDGVDFYTKVSRARFEELNADLFRSTLQPVEKSLTDAKMDKSLIHDIVMVGGSTRIPKVQS

Hsp70 B.ole (277) EATIEIDALYEGVDFYTKVSRARFEELCADLFRQTLDPVEKALNDAKMDKNQIHDIVLVGGSTRIPKVQS

Hsp70Ab A.aeg (277) EATIEIDALIDGIDYYTKISRARFEELCSDLFRSTLQPVEKALSDAKMDKRSIHDIVLVGGSTRIPKVQN

Hsp70 B.mor (277) EATIEIDALYEGIDFYTRVSRARFEELNADLFRGTLEPVEKALKDAKLDKSQIHDVVLVGGSTRIPKVQT

Hsp70 M.bra (277) EATIEIDALYEGIDFYTRVSRARFEELCADLFRGTLDPVEKALKDAKMDKSQIHDVVLVGGSTRIPKVQS

Hsp70 R.pom (277) EATIEIDALYEGVDFYTKISRARFEEMCGDLFRSTLDPVEKALNDAKMDKSQIHDIVLVGGSTRIPKVQS

Hsp70 L.mig (280) QASIEIDSLYEGIDFYTSITRARFEELNADLFRSTMEPVEKALRDAKMDKAQIHDIVLVGGSTRIPKVQK

Hsp70 L.sat (277) EATIEVDALYEGVDFYTKVSRARFEELCADLFRSTLEPVEKALNDAKMDKNQIHDIVLVGGSTRIPKVQN

Hsp70 D.moj (277) EATIEIDALFEGHDFYTKVSRARFEELCGDLFRGTLQPVEKALNDAKMDKSQIHDIVLVGGSTRIPKVQS

Hsp70 D.vir (277) EATIEVDALFEGHDFYTKVSRARFEELCADLFRNTLAPVEKALNDAKMDKQQIHDIVLVGGSTRIPKVQS

Hsp70 D.ana (277) EATIEIDALFEGHDFYTKVSRARFEELCADLFRNTLRPVEKALTDAKMDKGQIHDIVLVGGSTRIPKVQS

Hsp70Aa D.mel (277) EATIEIDALFEGQDFYTKVSRARFEELCADLFRNTLQPVEKALNDAKMDKGQIHDIVLVGGSTRIPKVQS

Hsp70 D.ant (277) EATIEIDALYEGVDFYTKVSRARFEELCADLFRNTLQPVEKALNDAKMDKNQIHDIVLVGGSTRIPKVQN

** * * * * ** ****** **** * **** * *** ** *** * ***********

351 ▼ 420

Hsp70S1 S.sin (347) MLQNYFNGK**SLNLSINPDEAVAYGAAIQAAILSGDK**SSKIQDVLLVDVAPLSLGIETAGGVMTKIVERNS

Hsp70P1 O.par (347) LLQNFFNGK**SLNLSINPDEAVAYGAAVQAAILSGDK**SSKIQDVLLVDVAPLSLGIETAGGVMTKIVERNS

Hsp68 O.par (347) LLQNFFNGKTLNLSINPDEAVAYGAAIQAAILSGDQSSKIQDVLLVDVAPLSLGIETAGGVMTKIVERNS

Hsp70 B.ole (347) LLQSFFCGKSLNLSINPDEAVAYGAAVQAAILSGDKSSEIQDVLLVDVAPLSLGIETAGGVMAKIVERNC

Hsp70Ab A.aeg (347) LLQNFFCGKSLNLSINPDEAVAYGAAVQAAILSGDKDEKIQDVLLVDVAPLSLGIETAGGVMTKLIERNS

Hsp70 B.mor (347) MLQNFFCGKKLNLSINPDEAVAYGAAVQAAILSGETDSKIQDVLLVDVAPLSLGIETAGGVMTKIIERNS

Hsp70 M.bra (347) LLQNFFCGKKLNLSINPDEAVAYGAAVQAAILSGEQDAKIQDVLLVDVAPLSLGIETAGGVMTKIIERNS

Hsp70 R.pom (347) LLQSFFGGKSLNLSINPDEAVAYGAAIQAAILSGDKSSQIQDVLLVDVAPLSLGIETAGGVMTKLIERNS

Hsp70 L.mig (350) LLQDFFNGKELNKSINPDEAVAYGAAVQAAILAGDKSEEVQDLLLLDVTPLSLGIETAGGVMTTLIKRNT

Hsp70 L.sat (347) LLQSFFCGKSLNLSINPDEAVAYGAAIQAAILSGDKSSEIQDVLLVDVAPLSLGIETAGGVMTKLIERNS

Hsp70 D.moj (347) LLQNFFSGKSLNLSINPDEAVAYGAAIQAAILSGDQSSQIKDVLLVDVAPLSLGIETAGGVMTKLIERNS

Hsp70 D.vir (347) LLQQFFGGKSLNLSINPDEAVAYGAAVQAAILSGDQSGKIQDVLLVDVAPLSLGIETAGGVMTKLIERNS

Hsp70 D.ana (347) LLQQFFHGKSLNLSINPDEAVAYGAAVQAAILSGDQTGKVQDVLLVDVAPLSLGIETAGGVMTKLIERNC

Hsp70Aa D.mel (347) LLQDFFHGKNLNLSINPDEAVAYGAAVQAAILSGDQSGKIQDVLLVDVAPLSLGIETAGGVMTKLIERNC

Hsp70 D.ant (347) LLQQFFCGKSLNLSINPDEAVAYGAAIQAAILSGDKSSAIQDVLLVDVAPLSLGIETAGGVMTKLIERNS

** * ** ** ************* ***** * * ** ** ************* **

421 490

Hsp70S1 S.sin (417) RIPCK**QTQTFTTYSDNQPAVTVQVFEGERAMTKDNNLLGTFNLTGIPPAPR**GVPKVEVTFDLDANGILNV

Hsp70P1 O.par (417) RIPCK**QSQTFTTYSDNQPAVTIQVFEGERAMTKDNNLLGTFNLTGIPPAPRGVPKIDVTFDLDANGILNV**

Hsp68 O.par (417) RIPCKQTQTFTTYSDNQPAVTIQVFEGERAMTKDNNLLGTFDLTGIPPAPRGVPKIEVTFDLDANGILNV

Hsp70 B.ole (417) RIPCKQTQTFSTYSDNQSGVTIQVYEGERVMTKDNNRLGTFDLSGIPPAPRGVPQIEVTFDLDANGILNV

Hsp70Ab A.aeg (417) RIPCKQTQIFSTYADNQPGVSIQVFEGERAMTRDNNRLGQFDLSGIPPAPRGVPQIEVTFDLDANGILNV

Hsp70 B.mor (417) KIPCKQSQTFTTYSDNQPAVTIQVYEGERAMTKDNNLLGTFDLTGIPPAPRGVPKIDVTFDMDANGILNV

Hsp70 M.bra (417) KIPCKQSQTFTTYSDNQPAVTIQVYEGERAMTKDNNLLGTFDLTGIPPAPRGVPKIDVTFDLDANGILNV

Hsp70 R.pom (417) RIPCKQSKTFTTYADNQPAVTIQVFEGERAMTKDNNLLGTFNLTGIPPAPRGVPKIDVTFDLDANGILNV

Hsp70 L.mig (420) TIPTKQTQTFTTYSDNQPGVLIQVYEGERAMTKDNNLLGKFELTGIPPAPRGVPQIEVTFDIDANGILNV

Hsp70 L.sat (417) RIPSKQTKTFTTYADNQPAVTIQVFEGERAMTKDNNMLGTFNLTGIPPAPRGVPKVDVTFDLDANGILNV

Hsp70 D.moj (417) RIPCKQSKTFTTYADNQPAVTIQVFEGERAMTKDNNVLGTFNLTGIPPAPRGVPKVEVTFDLDANGILNV

Hsp70 D.vir (417) RIPCKQTKTFSTYSDNQPGVSIQVYEGERALTQHNNSLGTFDLSGIPPAPRGVPQIEVTFDMDANGILNV

Hsp70 D.ana (417) RIPCKQTKTFSTYADNQPGVSIQVYEGERAMTKDNNALGTFDLSGIPPAPRGVPQIEVTFDMDANGILNV

Hsp70Aa D.mel (417) RIPCKQTKTFSTYADNQPGVSIQVYEGERAMTKDNNALGTFDLSGIPPAPRGVPQIEVTFDLDANGILNV

Hsp70 D.ant (417) RIPCKQSKTFTTYADNQPAVTIQVFEGERAMTKDNNLLGTFNLTGIPPAPRGVPKIDVTFDLDANGILNV

** ** * ** *** * ** **** * ** ** * * ********** **** ********

491▼ ▼ ▼ ▼ ▼ 560

Hsp70S1 S.sin (487) SAKDTSTGNSKNITIKNDKGRLSQAEIDKMLAEAERYAEEDEKQRQRVAAR**NQLEGYVFNVK**QSVEDAG-

Hsp70P1 O.par (487) **SAKDTSTGNSK**NITIKNDKGRLSQSDIDRMLAEAERYAEEDEKQRQRVAAR**NQLEGYVFNLKQSVEDAG-**

Hsp68 O.par (487) SAKELGTGNAKNITIKNDKGRLSQAEIERMLNEAEQYKDEDEQQRQRVTSKNALEGYVFSLKQAVEDAG-

Hsp70 B.ole (487) SAKELSSRNAKNITIKNDKGRLSQEDIDRMVNEAERYAEEDERQRNKIAARNNLESYVFGVKQALDGAG-

Hsp70Ab A.aeg (487) SAKEKSTGKEKNITIKNDKGRLSQADIDRMVSDAEKYREEDEKQRQRVSARNQLEGYCFQLKQSLESAG-

Hsp70 B.mor (487) SAKENSTGRSKNIVIKNDKGRLSQAEIDRMLSEAERYKEEDEKQRQRVAARNQLELYLFSVKQALDEAG-

Hsp70 M.bra (487) SAKENSTGRSKNIVIKNDKGRLSQAEIERMLAEAERYKDEDEKQRQRVAARNQLEAYVFSVKQALDDAAA

Hsp70 R.pom (487) TAKEMSTGNAKNIVIKNDKGRLSQADIDRMVNEAEKYAEEDEKQRQRVAARNQLESYVFNVKQAADEAG-

Hsp70 L.mig (490) TAVEKSTGKENKITITNDKGRLSKEEIERMVNEAERYRAEDEKQKATIAAKNGLESYCFNMKSTVEDEKL

Hsp70 L.sat (487) TAKEMSTGNAKNITIKNDKGRLSQADIDRMVSEAEKYAEEDEKHRQRIAARNQLEGYVFNVKQVVEDAG-

Hsp70 D.moj (487) TAKEMSTGNAKNITIKNDKGRLSQADIDRMVSEAEQYAEEDEKHRQRIAARNQLESYIFGVKEAAENAK-

Hsp70 D.vir (487) TAKEMSTGKAKNITIKNDKGRLSQAEIDRMVNEAERYADEDEKHRERITARNSLESYVFGVKQAVEQASP

Hsp70 D.ana (487) TAKEMSTGKAKNITIKNDKGRLSQAEIDRMVNEAERYADEDEKQRQRISSRNSLESYVFNVKQAVEQAGS

Hsp70Aa D.mel (487) SAKEMSTGKAKNITIKNDKGRLSQAEIDRMVNEAEKYADEDEKHRQRITSRNALESYVFNVKQAVEQAPA

Hsp70 D.ant (487) NAKEMSTGNVKNIVIKNDKGRLSQAEIDRMVNEAEQYADEDEKYRQRIAARNQLETYVFGVKQTVDQAG-

* * * ******* * * ** * *** * ** * * *

561 ▼ ▼ ▼ 630

Hsp70S1 S.sin (556) -DKLSQSDKNTVQKACEDTIKWLDNNNLADK**EEFEHRMQELTRQCSPIMTK**LHTG-GAQPQGG-------

Hsp70P1 O.par (556) **-SK**LSPEDKDTVLKSCDDTIKWLDNNNLAEK**EEFEHRFQELTRKCSPIMTK**LHSGSGAGQQGP-------

Hsp68 O.par (556) -DKLTQSDKDTVIKCCEETIKWLDANTLAEKDEYEHRQKEVTRVCSPIMTKLHGG---GPAGA-------

Hsp70 B.ole (556) -DKLSAQEKGEALKVCDDTIKWLDANTLADKEEYEDKMNTLTKLCSPIMTKLHSGGAQGAS---------

Hsp70Ab A.aeg (556) -DKLSESDKNTVKDKCDETLRWLDGNTMAEKDEFEHKMQELSRVCSPIMTRLHQGGTAGAGAS-------

Hsp70 B.mor (556) -DKLSDADKSTARDACDEALRWLDNNTLADQDEYEHKLKDVQRVCSPVMSKMHGAAPGGMPGG-------

Hsp70 M.bra (557) ADKLSEQDKSTARSACDEALKWLDNNTLAEQEEYEHRLKDVQRVCSPIMSKMHGAGAGG-----------

Hsp70 R.pom (556) -SKISQSDKDRVLEKCSETIKWLDANTTAEKEEFEYKLEELTKICSPIMTKMHQEAGAGPQP-------S

Hsp70 L.mig (560) KDKISDSDKQTILDKCNEVIRWLDANQLAEKEEFEEKQKELEQICNPIITKLYQGAGGAPGGMPGGFPGG

Hsp70 L.sat (556) -AKLSEADKNKILEKCNETIKWLDNNTTAEKEEYDYKYEEVSKFCTPITAKMHQQQQQTSG--------N

Hsp70 D.moj (556) -DKISQSERSTVLDKCSEAIKWLDANTTAEKEEYEYKLQELTKVCSPVMTKMHQSAGGGDGP-QA----S

Hsp70 D.vir (557) -DKLSDSDKSSVLDKCSETVKWLDANTTADKEEFEYKLKELTQHCSPIMTKLHQQ----G-QPQG---NA

Hsp70 D.ana (557) -DKLSEADKSSVLDKCNESIRWLDTNTTAEKEEFDHKLEELTRHCSPIMTKMHQQSAGAAGAPGG--PGA

Hsp70Aa D.mel (557) -GKLDEADKNSVLDKCNDTIRWLDSNTTAEKEEFDHKMEELTRHCSPIMTKMHQQGAGAAGGPG-----A

Hsp70 D.ant (556) -DKIPKSDKDRLMEKCSETINWLDNNTTAEKEEYDYKLEELTKICQPIMTRMHQQANGGAGPQPG----S

* * *** * * * * *

631 655

Hsp70S1 S.sin (617) SCGQQAGGFG---GGRSGPTVEEVD

Hsp70P1 O.par (618) SCGQQTGGFG---GARSGPTVEEVD

Hsp68 O.par (615) NCGQQAG------GARTGPTVEEVD

Hsp70 B.ole (616) -CGQQAGGFSGR----TGPTVEEVD

Hsp70Ab A.aeg (618) SCGQQAGGFGG----RTGPTVEEVD

Hsp70 B.mor (618) MPGGMPGGYQQAR--SDGPTVEEVD

Hsp70 M.bra (616) -PGGMPGGYGNHQQ-NSGPNVEEVD

Hsp70 R.pom (618) NCGQQSGNFAGG--QYGGPTVEEVD

Hsp70 L.mig (630) FPGAGGAAAGGAGAGGAGPTIEEVD

Hsp70 L.sat (617) NCGQQAG-FGGG--NYEGPTVEEVD

Hsp70 D.moj (620) HCGQQARGCNGS----GGPTIEEVD

Hsp70 D.vir (618) NCGQQAGGFGGAGG-YQGPTVEEVD

Hsp70 D.ana (624) GCGQQAGGFG---G-YSGPTVEEVD

Hsp70Aa D.mel (621) NCGQQAGGFG---G-YSGPTVEEVD

Hsp70 D.ant (621) NCSQQAGGFGSG--SYSGPTVEEVD

** ****

S.sin. – *Stratiomys singularior*, O.par – *Oxycera pardalina*, B.ole – *Bactrocera oleae* (acc. no. CAI44197); A.aeg – *Aedes aegypti* (acc. no. ACJ64193), B.mor – *Bombix mori* (acc. no. BAF69068), M.bra – *Mamestra brassicae* (acc. no. BAF03555), R.pom – *Rhagoletis pomonella* (acc. no. ABL06948), L.mig – *Locusta migratoria* (acc. no. AAO21473), L.sat *– Liriomyza sativae* (acc. no. AAW32099), D.moj – *Drosophila mojavensis* (acc. no. XP_001998792), D.vir – *Drosophila virilis* (acc. no. XP_002058650), D.ana – *Drosophila ananassae* (acc. no. XP_001953834), D.mel – *Drosophila melanogaster* (acc. no. AAG26887), D.ant – *Delia antique* (acc. no. AAY28732). The position of serine residue in the ATPase domain is shadowed. Black arrows above the aligned sequences indicate amino acid residues specific to Hsps of the both Stratiomyidae species or polymorphisms common with other highly thermotolerant organisms (e.g. *L. migratoria*).
