# Supplementary material for: Organization and evolution of hsp70 clusters strikingly differ in two species of Stratiomyidae (Diptera) inhabiting thermally contrasting environments
Source: BMC Evol Biol. 2011 Mar 22;11:74. doi: 10.1186/1471-2148-11-74 (PMC3071340; doi:10.1186/1471-2148-11-74)
Supplement: Additional file 15 — Table S3: Primers for PCR and RACEs, used in the study. [file 1471-2148-11-74-S15.DOC]

**Additional file 15: Table S3. Primers for PCR and RACEs, used in the study.** Table containing all primer sequences used for sequencing individual genes belonging to *hsp70* family of *S. singularior* and *O. pardalina* as well as in 5’RACE experiments.

| Primer sequence (5’ – 3’) | Localization, orientation and application |
| --- | --- |
| TGTTCTTCGGGTTCATAGCCAC | 5’-end of *S. singularior hsp70* gene, outward |
| ATAATGACGAAGTTGCACACCG | 3’-end of *S.singularior hsp70* gene, outward |
| CTGGTTCTTGGCCGCGTCG | 5’-end of *O. pardalina hsp70* gene, outward |
| GAATGGAACCGAGATGCTGTG | 3’-end of *O. pardalina hsp70* gene, outward |
| ACCGACTAAAACCACCTCCATA | 5’-end of *O. pardalina hsp68* gene, outward |
| CCAACGACCAGGGAAACAGG | 5’-end of *O. pardalina hsp70* gene, inward |
| ACACGTAGCCTTCGAGCTGG | 3’-end of *O. pardalina hsp70* gene, inward |
| TGTTCTTCGGGTTCATAGCCAC | *S. singularior hsp70*, 5’-RACE-1 |
| CAATTCCTCAAGTCCTCCTG | *S. singularior hsp70*, 5’-RACE-2 |
| ATAATGACGAAGTTGCACACCG | *S. singularior hsp70*, 3’-RACE-1 |
| CAATGCAGTCCGATAATGACGA | *S. singularior hsp70*, 3’-RACE-2 |
| TCCGGCCCGACAGTGGAGGA | *O. pardalina hsp70*, 3’-RACE-1 |
| GACAACAATAACCTGGCGGAGA | *O. pardalina hsp70*, 3’-RACE-2 |
| CTGGTTCTTGGCCGCGTCG | *O. pardalina hsp70*, 5’-RACE-1 |
| CCTCTTAGCGTCGAACACCG | *O. pardalina hsp70*, 5’-RACE-2 |
| CCGACAGAAAGAAGTAACGAGA | *O. pardalina hsp68*, 3’-RACE-1 |
| AAGGACGAGTACGAACACCGA | *O. pardalina hsp68*, 3’-RACE-2 |
| GCGGTTTCCCTGGTCGTTCG | *O. pardalina hsp68*, 5’-RACE-1 |
| CATCCCCGATCAACCGTTCTG | *O. pardalina hsp68*, 5’-RACE-2 |
